# Designing mobile application messages to impact route choice: A survey and simulation study

**DOI:** 10.1371/journal.pone.0284540

**Published:** 2023-04-20

**Authors:** Christina Maria Mayr, Anne Templeton, Gerta Köster

**Affiliations:** 1 Department of Computer Science and Mathematics, Hochschule München University of Applied Sciences, Munich, Germany; 2 Department of Informatics, Technical University of Munich, Garching, Germany; 3 School of Psychology and Language Sciences, University of Edinburgh, Edinburgh, United Kingdom; Qatar University, QATAR

## Abstract

Crowd congestion is a common issue at train stations around major sports events, and puts passengers at risk and lowers service quality. Guiding arriving fans along less traveled routes may alleviate congestion. Smartphone apps provide a medium to deliver route suggestions but the messages they provide are pivotal to adherence. We explore how message design affects pedestrians’ willingness to follow route instructions. We present an online survey conducted with two groups: football fans, and students and faculty associates. We vary the presence of top down views of the route choices at train station Münchner Freiheit in Munich, real-time information on congestion, and appeals to team spirit. We compute a distribution of route choices that suggests that congestion may be reduced with the right combination of message components for each target group. We then use a computer simulation to investigate the congestion situation. Our results suggest that lowest congestion is achieved when people base their decisions on real-time information. The social identity approach is highlighted in our study as having a possible influence on message design. Moreover, it indicates that the implementation of such apps in real-life applications can improve safety. Our methodology can be applied to other scenarios to test the suitability of apps and message designs.

## Introduction

### Motivation

Major sports events such as football matches put public transportation hubs under strain. Crowds accumulate before and after the game, a situation which traffic providers try to mitigate by providing more frequent train service. However, even if the station capacity were, in theory, sufficient to keep pedestrian streams flowing, certain routes and hot-spots regularly jam. One idea to alleviate this is to redirect pedestrians to less used routes and to employ an app to convey this information.

Often, traffic apps for public transport show the quickest route to a destination [1], and how busy a particular means of transport is. For example, the German rail provider Deutsche Bahn indicates the capacity utilization of its trains [2]. Munich’s public transport system does the same for its buses [3]. At train stations, similar information is becoming available, e.g. when elevators or escalators are out of service [4].

It is more difficult to inform, in a meaningful way, on congestion inside a station because occupancy fluctuates with the arrivals and departures of trains. In order to have a positive effect on pedestrian distribution, the information would have to adapt dynamically to the situation and travelers would have to be willing to follow suggestions. This challenge is the motivation behind the combined online survey and traffic simulation study that we present in this manuscript.

### Related work

Communication sciences investigate the effect of how an information is provided on peoples’ reaction to it. Both, quantity and quality play a role: how much information is necessary? In which way should a certain piece of information be presented? The latter includes the so-called message framing, i.e., ‘presenting logically equivalent options in semantically different ways’ [5]. Message framing plays a role in numerous fields of application [6]. In health sciences, it is investigated how messages should be phrased to help individuals improve their health status. Examples are advice on diet [7], hygiene [8], vaccination [9], or giving up smoking [10]. In marketing, suitable framing is supposed to increase sales [11, 12]. Political communication tries to win over voters through framing strategies [13, 14]. Many of these studies focus on gain or loss, e.g., Nobel [10] investigates how gain-framed messages (‘if you quit smoking, you will live longer’) and loss-framed messages (‘if you continue to smoke you will lose money’) affect peoples’ success in quitting smoking. Other studies vary the amount of information and number of instructions [15–18]. Message framing has also been applied to emergency situations, for example in Carter et al’s. [17, 18] study on mass decontamination which found that the highest level of public compliance was achieved when they were provided with both health-focused information and practical information.

Research within the social identity approach [19], suggests that crowd behavior is influenced by the extent to which the people in a crowd have a shared social identity, i.e., all feel part of the same social group. For example, there is greater coordination in psychological crowds where people share a social identity compared to physical crowds without a shared social identity [20]. In addition, psychological crowd members are more influenced by members of their social group (in-group members) than people who are in opposing social groups (out-group members) or whose group membership is not known [18, 21–23]. Psychological crowd members also prefer to stay in denser and more central parts of the crowd because they associate it with a positive experience [24], and will regulate their speed and distance to stay in close physical proximity to in-group members [25, 26]. Such behaviors can change the speed-density relationship, e.g. pilgrims at the Kumbh Mela festival have high speeds even at high densities [27].

We argue, that since social identity can affect behavior it should be considered in communication of route suggestions [28, 29]. From this we derive two research questions: how should route recommendations be designed to redirect football fans who share a social identity? How does this design affect pedestrian traffic at traffic hubs?

To investigate the traffic situation, we propose to use computer simulations. In prior simulation studies [30], we showed that a simple route recommendation system can alleviate congestion even when not everybody adheres to the route recommendation. We did this, by varying compliance rates in a simple traffic scenario that was also inspired by the train station Münchner Freiheit. However, we omitted the crucial link how message design translates into different compliance behaviors which we will examine in this study.

### Goal and overview of this work

**Fig 1:**
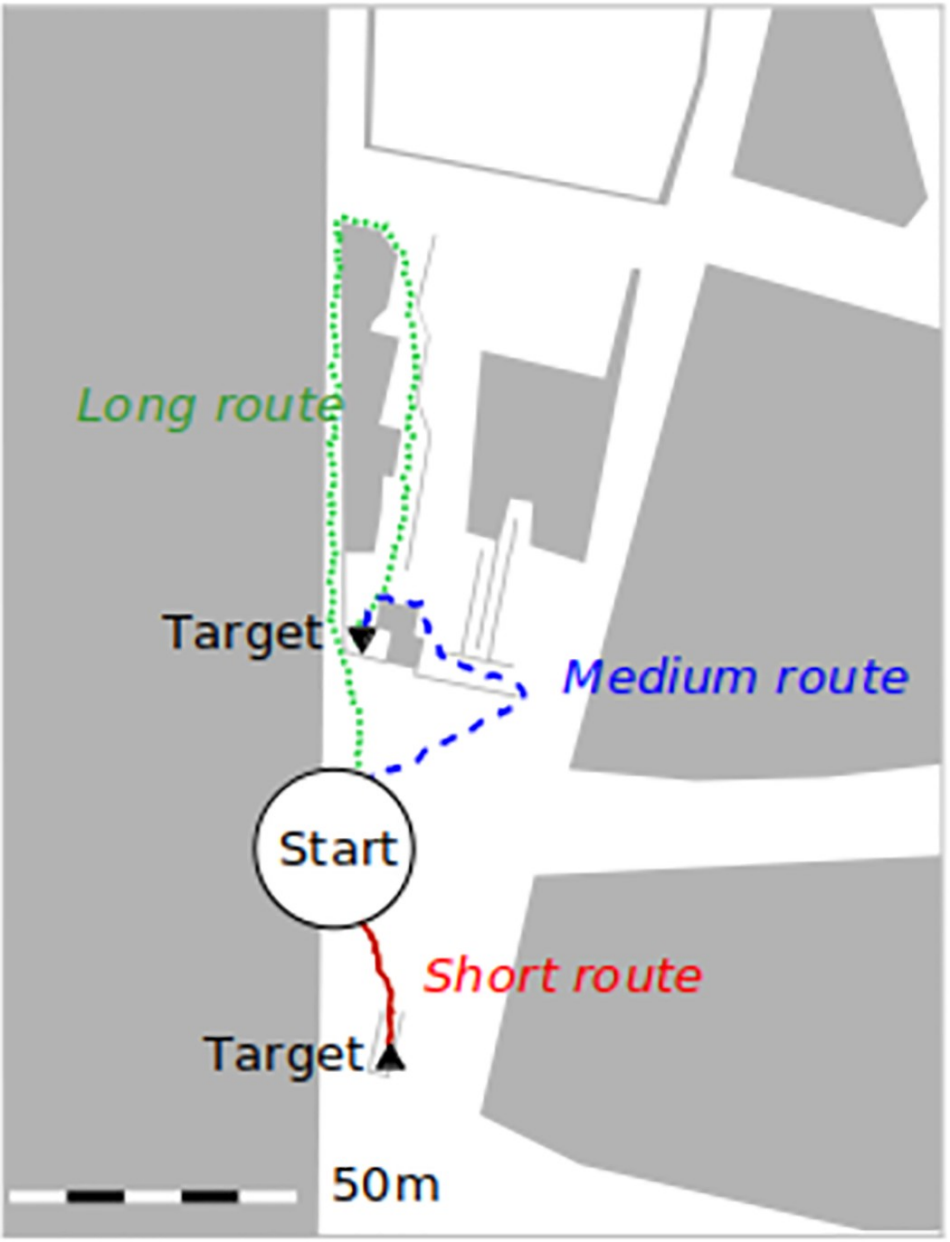
Surface level of the train station Münchner Freiheit in Munich [30]. We took the snapshot from the *Vadere* simulator’s graphical user interface. We used OpenStreetMap to understand the topography of the train station and then recreated this for the simulation in the Vadere simulator’s graphical user interface. Three main routes lead to the trains of which the vast majority of people use the short route. In our simulation study we assume that up to 300 fans per minute arrive at the station which makes them experience congestion on their way to the train.

Our goal is to investigate how the design of messages in a navigation app affects pedestrians’ route choice and, thus, congestion. Our use case is a real-life situation: we look at the train station Münchner Freiheit in Munich, see Fig 1. We consider eight different message designs that all recommend a certain route, but contain different components to convey the route recommendation. One component is real-time congestion information where congestion is highlighted in red on a simplified map. Another one marks the route from a bird’s eye view. The third one is a motivational phrase that appeals to the team spirit of football fans. We expect responses to depend on message framing but also on the level of social identification, i.e., whether they identify with the group or not. That is why we test two groups: football fans who we expect to have a higher level of shared social identification and a control group who we expect to have a lower level of shared social identification.

The paper is organized as follows. The next three sections comprise the methodology that we propose: an online survey in the section *Survey*, in which we show our message designs to the participants and ask them to choose routes. In the section *Traffic assignment model*, we introduce a traffic assignment model that computes route probabilities based on the survey data. We use this model to inform a computer simulation in the section *Traffic simulation*. In the simulation study we assess the effect of the message design on the congestion situation. We discuss our findings in the *Discussion*. Then, we give a brief summary and provide a *Conclusion*.

## Survey

### Ethics statement

We obtained ethical approval from the Hochschule München University of Applied Sciences (Munich, Germany) before we conducted the survey. At the beginning of the survey, participants were informed about the research and told that their anonymised answers would be made publicly available. We also informed them that they were free to withdraw from the survey at any time. To participate in the survey, they had to confirm their consent and that they were at least 16 years old at the time of the participation.

### Setup

We conducted two identical online surveys to assess the effect of message design on the route choice in the scenario Münchner Freiheit (Fig 1): one with students and faculty associates at Hochschule München (Germany), and one with football fans of FC Bayern München. We addressed the students and faculty associates through Facebook, the Vadere research simulator webpage (www.vadere.org) and a general email to students and faculty associates at Hochschule München. We also sent personal appeals to the students of our department.

Football fans were recruited using the official FC Bayern fan group on Facebook (www.facebook.com/FCBFanbetreuung) and Twitter (www.twitter.com/FCBayern_FB). In total, 1365 out of 3067 (44.51%) participants completed the survey, while 49.12% dropped out in the introductory section, before or immediately after the declaration of consent. 444 out of the 1365 participants were students or faculty associates (43% female, 54% male). 921 participants were fans (18% female, 80% male). Many fans (72%) were between 26 and 50 years old, see Table 1. Students and faculty associates were younger: only 36% fell in the range from 26 to 50, half of them were younger than 26, and 14% were older than 50. Participation was voluntary and there were no monetary incentives. The survey for the students and faculty associates was conducted from 16.11.2021 to 23.02.2022. The survey for the fans ran from 24.02.2022 to 01.03.2022.

**Table 1:** Age distribution of two populations: Football fans (1), and students and faculty associates (2).

Group	<18	18–25	26–35	36–50	51–65	>65	n
Fans	0%	17%	38%	34%	10%	1%	921
Students and faculty associates	7%	43%	28%	8%	11%	3%	444

Students and faculty associates are younger than fans which might be explained by the fact that most of them are students.

**Fig 2:**
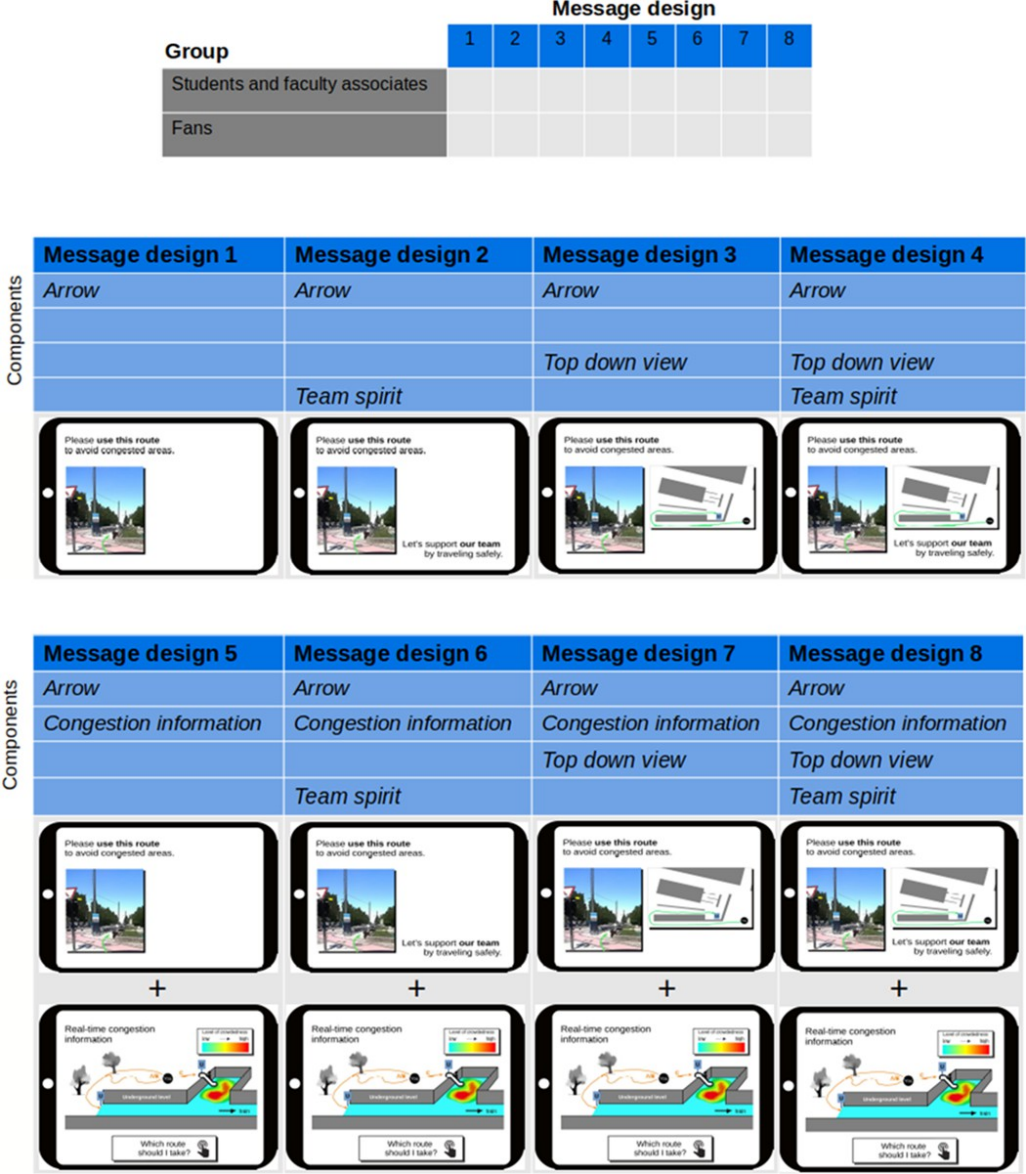
Experimental between-subject design. We obtained eight message levels by combining three optional message components: *congestion information*, *top down view*, and *team spirit*. For a detailed view of the message components see Fig 3.

**Fig 3:**
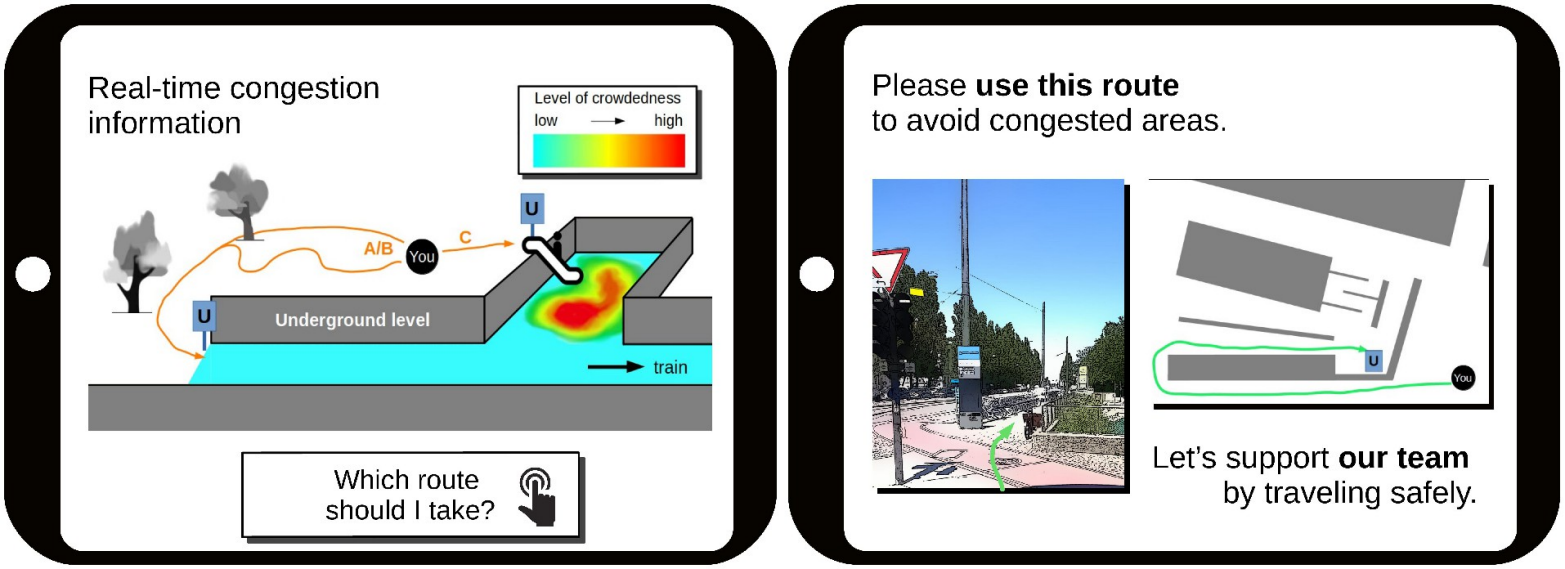
Message design with all possible message components. Right: each message design contains the sentence ‘Please use this route […]’ and a picture with an *arrow* pointing to the entrance of the recommended route. The components *top down view* and *team spirit* (‘Let’s support […]’) are optional. For message designs without *congestion information*, only the screen on the right is displayed to the participant. For designs with *congestion information* included, both screens are displayed. We imagine that in a real-life application, the left screen is what the app user sees first, followed by the second screen that appears when clicking on “Which route should I take?” (left). In the survey, we placed the screens next to each other.

We used a mixed between- and within-subjects design. The between-subjects design has one factor, message design, with eight levels, see Fig 2. The levels stem from the combination of components. The first component is real-time *congestion information* where congestion is highlighted through a color scheme. The second is a *top down view* that depicts the underground train station from a top down view and with the recommended route marked in green. The third component is labeled *team spirit* and refers to the motivational phrase ‘Let’s support our team by traveling safely’. The combination of these three components leads to 2^3^ = 8 message designs in total. Regardless of the combination, every message design contains a picture with an *arrow* that points in the direction of the long route and a route recommendation that is phrased as follows: ‘Please use this route to avoid congestion.’. Fig 3 depicts the design with all optional components.

The within-subjects design has one factor, information provision, with two levels: *prior to information*, and *after receiving information*. We collect quantitative self-report data from a questionnaire. The dependent variables were: route attractiveness (‘How likely is it that you take the following routes?’), and social identification (‘I can imagine being part of the fan community.’). For each, responses are scored on a 5-point Likert scale (1 = Very unlikely, 2 = Unlikely, 3 = Neutral, 4 = Likely, 5 = Very likely).

To conduct the survey, we used the free and open source online survey tool *LimeSurvey*. See www.limesurvey.org for more information. Conditions were randomly assigned to participants. Please find the survey setup and the survey results in the S1 Appendix, S1 Table.

### Survey results and discussion

**Fig 4:**
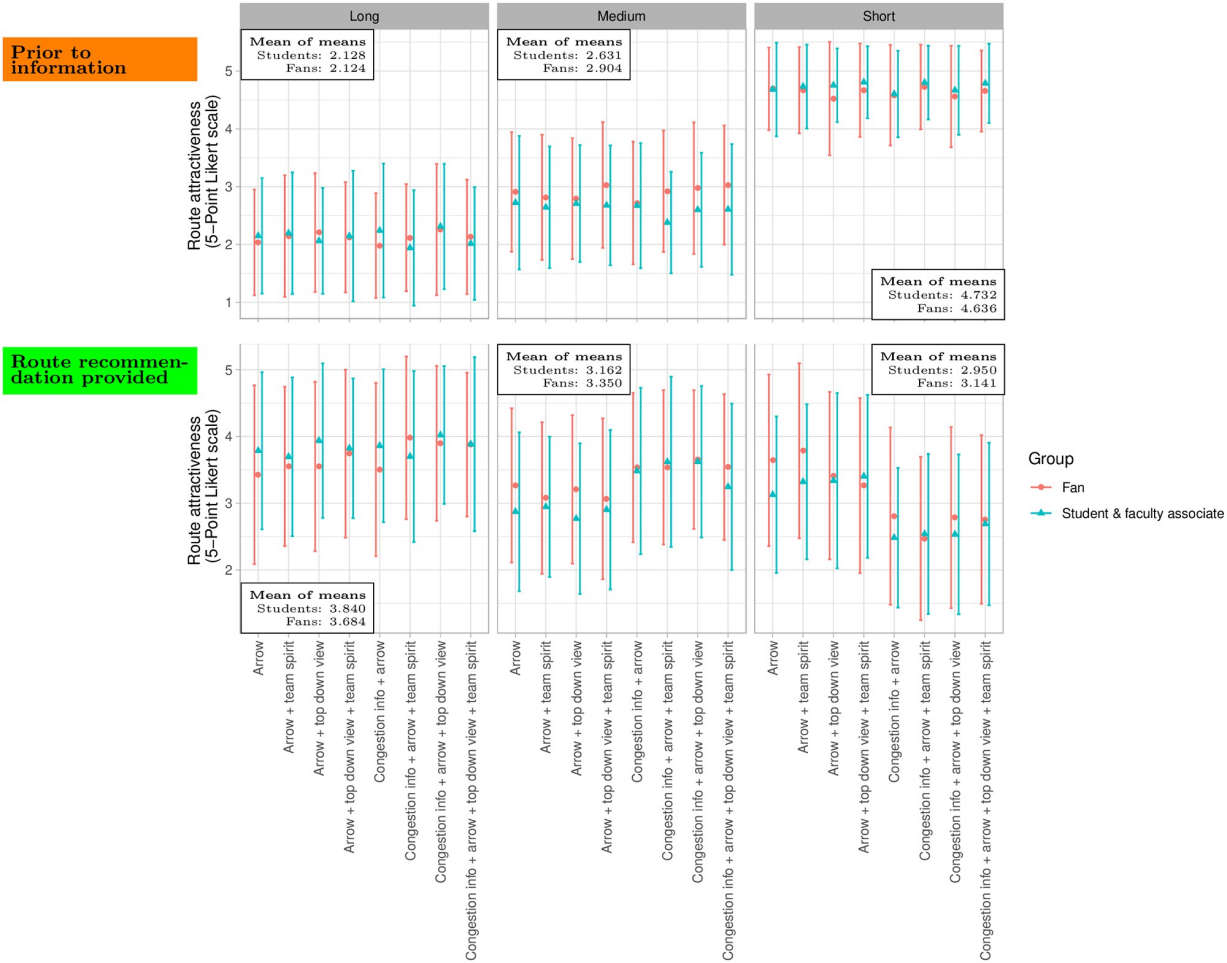
Attractiveness of routes expressed through the Likert scale value assigned by participants. The error bars around the mean values (dots) represent the standard deviations. Prior to information, students and faculty associates as well as fans had a clear route preference: they favored the short route (top right) over the medium route (top center) followed by the long route (top left). With information provision, the short route became less attractive (bottom right) compared to the setting without information (top right), while the long route became more attractive (left).

We observed that, prior to information, both fans as well as students and faculty associates selected their routes according to path lengths. They favored the short route overall, see Fig 4 and the (S2F Table). This behavior changed with the route recommendation: the long route became more attractive, and the short route less attractive (compare the top and bottom rows in Fig 4, see the S2C and S2D Table). As expected, Kruskal-Wallis tests revealed that the message design had a stronger effect on fans than on students and faculty associates: for the fans, the message design had a significant effect on the attractiveness of the short (*p* = 0.0000, *H* = 95.76, *df* = 7), medium (*p* = 0.0000, *H* = 33.04, *df* = 7) and long (*p* = 0.0022, *H* = 22.42, *df* = 7) route, while for the students and faculty associates, the design only made a significant difference for the short (*p* = 0.0000, *H* = 45.35, *df* = 7) and medium (*p* = 0.0000, *H* = 32.38, *df* = 7) route. The attractiveness of the long route was not affected (*p* = 0.8010, *H* = 3.8113, *df* = 7). See the (S2G Table). We conclude that football fans are more responsive to message design than students and faculty associates.

Interestingly, students and faculty associates seemed to be more willing to follow route recommendations: the mean attractiveness of the long route was 3.84 for the students and faculty associates and only 3.69 for the fans, see Table 2.

**Table 2:** Attractiveness of long route expressed through scores on a 5 point Likert scale.

Message design	Attractiveness of long route: mean values	
	Students and faculty associates	Fans
Congestion info + arrow	3.86 (n = 58)	3.50 (n = 103)
Congestion info + arrow + top down view	**4.02** (n = 45)	3.90 (n = 116)
Congestion info + arrow + team spirit	3.70 (n = 50)	**3.98** (n = 102)
Congestion info + arrow + top down view + team spirit	3.88 (n = 61)	3.88 (n = 107)
Arrow	3.79 (n = 47)	3.43 (n = 143)
Arrow + top down view	3.94 (n = 65)	3.55 (n = 111)
Arrow + team spirit	3.70 (n = 56)	3.55 (n = 103)
Arrow + top down view + team spirit	3.82 (n = 62)	3.74 (n = 136)
Average of means	3.84	3.69

We asked the participants how likely it is that they take the long route (1: very unlikely, 2: unlikely, …, 5: very likely). The higher the mean value, the more attractive the long route. The message design that contains all optional components (row 4) does not achieve the highest values: 4.02, 3.98.

We then conducted Mann-Whitney U tests to assess the effect of adding the message components *congestion information*, *top down view*, and *team spirit* on the attractiveness of each route. Please find an introduction to the Mann-Whitney U test and details on its implementation in the (S2 Appendix). We added one message component at each step, thus mitigating, to an extent, the inter-dependencies between designs.

We found that adding components did not always help direct people away from the short congested route, but it never had a negative effect: through adding components, the short route became less attractive (see Tables 3–5) or remained equally attractive (S2A and S2B Table). At the same time, the medium and long routes became more attractive (see Tables 3–5) or remained equally attractive (S2A and S2B Table). Crucially, adding all components together did not lead to an increased attractiveness of the long route, see Table 2. Hence, there is evidence for an interaction between the combinations. We also observed a varying influence of the components. The congestion information had always an effect on fans, and students and faculty associates: the attractiveness of at least one route changed (see Tables 3 and 4). The *top down view* and *team spirit* only had an effect in certain combinations with other message components. They also had a lower effect on route choice: either the short route became less attractive or the long route became more attractive. Both at the same time was never the case, see Table 5.

**Table 3:** Effect of adding congestion information to message designs on the route attractiveness for *students and faculty associates*.

Message design	Route attractiveness		
	Short	Medium	Long
Arrow	*p* = 0.0019, *W* = 1804.0 ⇓	*p* = 0.0117, *W* = 989.0 ⇑	*not significant (p > 0.05)*
Arrow + top down view	*p* = 0.0009, *W* = 1974.5 ⇓	*p* = 0.0003, *W* = 884.5 ⇑	*not significant (p > 0.05)*
Arrow + team spirit	*p* = 0.0010, *W* = 1895.5 ⇓	*p* = 0.0031, *W* = 949.5 ⇑	*not significant (p > 0.05)*
Arrow + top down view + team spirit	*p* = 0.0011, *W* = 2514.5 ⇓	*not significant (p > 0.05)*	*not significant (p > 0.05)*

For each message design, we used a Mann-Whitney U test to assess whether adding *congestion information* has an effect (*p* < 0.05) or not. Adding *congestion information* made the short route always significantly less attractive (⇓). The medium route became significantly more attractive (⇑) except in the case where all other message components were already present (bottom row). The attractiveness of the long route was not affected.

**Table 4:** Effect of adding congestion information to message designs on the route attractiveness for *football fans*.

Message design mann	Route attractiveness		
	Short	Medium	Long
Arrow	*p* = 0.0000, *W* = 9855 ⇓	*not significant (p > 0.05)*	*not significant (p > 0.05)*
Arrow + top down view	*p* = 0.0003, *W* = 8143 ⇓	*p* = 0.0021, *W* = 5006.5 ⇑	*p* = 0.0331, *W* = 5433 ⇑
Arrow + team spirit	*p* = 0.0000, *W* = 7940 ⇓	*p* = 0.0038, *W* = 4087.5 ⇑	*p* = 0.0047, *W* = 4107 ⇑
Arrow + top down view + team spirit	*p* = 0.0023, *W* = 8857.5 ⇓	*p* = 0.0022, *W* = 5678 ⇑	*not significant (p > 0.05)*

For each message design, we used a Mann-Whitney U test to assess whether adding *congestion information* has an effect (*p* < 0.05) or not. Adding *congestion information made* the short route always significantly less attractive (⇓). For some message designs, the medium and long route became significantly more attractive (⇑).

**Table 5:** Effect of adding a top down view or team spirit to message designs for *football fans*.

Added component	Message design	Route attractiveness		
		Short	Medium	Long
Top down view	Arrow + team spirit	*p* = 0.0024, *W* = 8555.5 ⇓	*not significant (p > 0.05)*	*not significant (p > 0.05)*
	Arrow + congestion info.	*not significant (p > 0.05)*	*not significant (p > 0.05)*	*p* = 0.0305, *W* = 5006 ⇑
Team spirit	Arrow + congestion info.	*not significant (p > 0.05)*	*not significant (p > 0.05)*	*p* = 0.0069, *W* = 4157 ⇑

For each message design, we used a Mann-Whitney U test to assess the effect of adding a *top down view* or *team spirit*, that is present, if *p* < 0.05. When adding a *top down view* to the message design with *arrow* and *team spirit* (first row), the short route became significantly less attractive (⇓). For the message design with *congestion information* (second row), the long route became significantly more attractive (⇑). The *team spirit* made the short route less attractive only in combination with the *arrow* and the *congestion information* (bottom row).

We discovered evidence for the influence of the social identity approach when appealing to the fan’s *team spirit*: adding *team spirit* had an effect on fans for one message design (see Table 5), but never on students and faculty associates (see S2A Table).

One explanation for this is that the team spirit manipulation only works when there is an existing team identity to manipulate. We therefore investigated how strongly the two groups could imagine being part of the group by asking ‘I can imagine being part of the fan community’. There was a significant difference between fans (mean = 3.859) and students and faculty associates (mean = 3.252), (*W* = 270588, *p* < 0.05). We observed a significant influence of the *team spirit* for one message design only. Nonetheless, we do not believe that this is a statistical coincidence. We argue that there are other factors that may mask the effect: adding increasing amounts of information may lead to information overload. Also the reaction may already have been strong before team spirit is added, since the addition of the *team spirit* and the *top down view* together did not make the long route more attractive. A Kruskal-Wallis test revealed that the long route is equally attractive for designs that contain –in addition to *congestion information*– a *top down view* (*n* = 116, mean = 3.897) or *team spirit* (*n* = 102, mean = 3.980) or both (*n* = 107, mean = 3.879) (*p* = 0.39, *H* = 1.884, *df* = 2). Please find an introduction to the Kruskal-Wallis test and details on its implementation in the (S2 Appendix). We conclude that the social identity can be influential, but it is very sensitive to environmental conditions and having a pre-existing social identity to prime.

## Traffic assignment model

We aimed to ascertain how message design affects safety, where safety is indicated by congestion. For that purpose, we estimated route choice proportions [31] that describe how people distribute across the different routes. With these, we hoped to better understand the effect of message design on the actual route choice. Furthermore, the route choice proportions are crucial parameters for the traffic simulation that we present in the next section. Before we present the route choice proportions for our scenario, we will introduce a novel traffic assignment model that we created to estimate route choice proportions based on survey data.

Our novel model uses the Likert scale survey data as input to compute route choice proportions for the short (*p*_1_) medium (*p*_2_), and long (*p*_3_) route. The basic idea is that the scores from the survey represent route frequencies. Imagine a survey participant who rated the shortest route at 4, the medium route at 3, and the long route at 3. She will pick the shortest route 4 out of 10(= 4 + 3 + 3) times, the medium 3 out of 10 times and the short 3 out of 10 times. We argue that this portrays the likely behavior in the long run. With this in mind, we define the individual route probability *p*_*j*,*m*_ that a participant *m* selects route *j* as

$$\begin{array}{c} p_{j,m}=\frac{l_{j,m}}{\sum_{j}l_{j,m}} \end{array}$$ (1)

where *l* is the route attractiveness that we take from the survey. Note that the division through ∑_*j*_ *l*_*j*,*m*_ achieves that the sum of probabilities equals 1: ∑_*j*_ *p*_*j*,*m*_ = 1. This normalization also automatically solves the problem that participants might interpret the scale differently, e.g. the route distribution is [1/3, 1/3, 1/3] no matter whether a participant rated the routes with [2, 2, 2] or [3, 3, 3].

We argue that the average of the single behaviors is a good characterization of the behavior of the population as a whole. Therefore, we define the route probability *p*_*j*_ for the whole population as

$$\begin{array}{c} p_{j}=\frac{1}{m}\sum_{m}p_{j,m} \end{array}$$ (2)

We interpret these route probabilities as frequencies that equal route choice proportions in a traffic assignment model. With our definition, route choice proportions depend on route attractiveness and thus on the message design.

For each group and for each message design, we computed the route choice proportions (Eqs 1 and 2) from the survey data, see Table 6. We observed that prior to any information, the route choice proportion of the short route was almost equal for both groups: 51% for the students and faculty associates, 49% for the fans. Similarly, only 22% of the students and faculty associates or the fans took the long route. This changed when information was provided. In all cases, fewer persons took the short route according to our traffic assignment model. Even in the case with the most sparse information, with just an arrow pointing in the direction of the long route, the percentage of people on the short route decreased from 49% to 35%, see Table 6. We think that this is in line with the survey results and, thus, that our traffic assignment model is suitable to inform a computer simulation of traffic.

**Table 6:** Route choice proportions after receiving information.

Group	Message design	Percentage of persons on route		
		*p*_1_ (short)	*p*_2_ (medium)	*p*_3_ (long)
Students	Congestion info + arrow	25	35	40
	Congestion info + arrow + top down view	25	35	40
	Congestion info + arrow + team spirit	26	36	38
	Congestion info + arrow + top down view + team spirit	28	32	40
	Arrow	32	29	39
	Arrow + top down view	33	27	40
	Arrow + team spirit	33	29	37
	Arrow + top down view + team spirit	34	28	38
Fans	Congestion info + arrow	29	36	35
	Congestion info + arrow + top down view	27	35	38
	Congestion info + arrow + team spirit	25	35	40
	Congestion info + arrow + top down view + team spirit	27	34	38
	Arrow	**35**	**31**	**33**
	Arrow + top down view	34	31	35
	Arrow + team spirit	36	29	35
	Arrow + top down view + team spirit	32	30	38

We proposed a simple traffic assignment model (see Eqs 1 and 2) that translates the Likert scores by which survey participants rate the attractiveness of a route into percentages of passengers *p*_*j*_ that would take that route. In the worst case (bold), only 33% of the fans take the long route, while 35% take the short route.

## Traffic simulation

As a final step, we used computer simulations to study the effect of the message design on crowd congestion. For that purpose, we simulated a route recommendation system that acts on a virtual crowd with which we studied the resulting traffic patterns. For our system we employed a simple logic that points virtual pedestrians to the long route when the other routes are crowded.

### Simulation model and implementation

Our simulation model included the mobility of pedestrians, their reaction to route recommendations, and the dynamic provision of rerouting messages.

**Fig 5:**
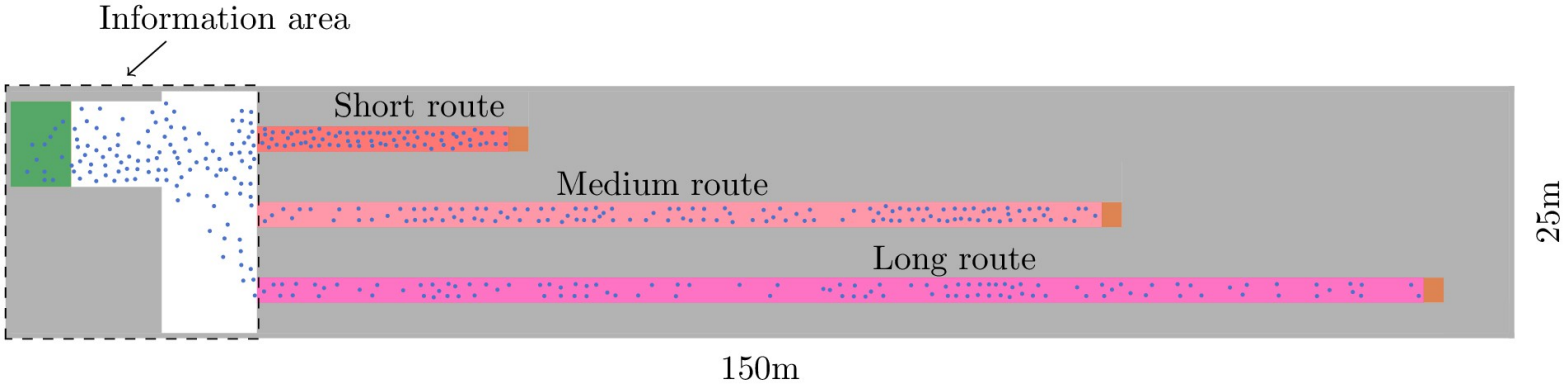
Simplified scenario with three routes of different lengths. They correspond to the three most important routes to the train platform at the train station Münchner Freiheit. Virtual pedestrians are generated in the (green) source on the left and walk to the (orange) destinations on the end of each corridor where they disappear. We imagine that they would enter the platform and then board trains. Without information about half of the virtual pedestrians take the short route. When the recommendation system is switched on it steers the crowd to the longest route within the information area whenever the density is the lowest within the long route.

To simulate the mobility of pedestrians, we used a validated pedestrian locomotion model, namely, the Optimal Steps Model [32]. The Optimal Steps Model is a microscopic model where each virtual pedestrian is modeled individually. At each step, the virtual pedestrians minimize their distance to the target while skirting others and obstacles such as walls. In our simulation, 300 virtual pedestrians per minute spawn at the source (Fig 5, left) and walk to the destination (Fig 5, right) along three paths. These correspond to the three most important routes from the bus to the train platform at the Münchner Freiheit, see Fig 1. When virtual pedestrians reach the end of a corridor (right), they disappear from the simulation which in reality would correspond to boarding the train. Without any route recommendation provided, 49% of the virtual pedestrians take the short route, 29% take the medium route, and 22% take the long route in the fan scenario. In the scenario with students and faculty associates, the route distribution is slightly different: 51% (short), 27% (medium), and 22% (long). Note that these are the route choice proportions that we computed in the previous section. Due to the higher occupancy of the short route, congestion occurs in the simulation in front of the entrance of the short corridor, see Fig 5. In reality, we observed a similar situation at the train station in front of the escalator in the short route. This is why we find that the main characteristics of the locomotion behavior are captured by the topography model despite its simplicity, see Fig 5. Also, its simplicity allows us to concentrate on the behavior of the overall system while saving computational cost.

Route recommendations provided via a mobile phone application have the advantage that they can be easily adjusted depending on the current traffic situation, e.g., the long route is recommended only when the short route is congested. If the long route were to be recommended even though another route is free, this could lead to a loss of trust which could reduce the compliance to follow route recommendations. Therefore, we argue that redirection measures should only be provided when necessary. Hence, we introduced a simple heuristic to decide when to recommend the long route: every 2*s*, we count the virtual pedestrians in the corridors, see Fig 5. We recommend the long route only, when it is less congested than the other routes, i.e., the pedestrian density is the lowest within the long corridor. Otherwise, no information is provided.

When people receive route recommendations, the distribution of people over the three routes changes. We use the route choice proportions that we computed in the previous section, see Table 6. If virtual pedestrians receive a route recommendation, their new route is drawn from a random distribution whose probabilities equal the route choice proportions from Table 6. We assume that the number of virtual pedestrians is high enough to represent the route distribution sufficiently accurately. Note that the route distributions depend on the target group and the message design. For example, fans that receive route recommendations, have the following route distribution: 35% (short), 31% (medium), 33% (long). If the same message design is displayed to students and faculty associates, the route distribution is different: 32% (short), 29% (medium), 39% (long).

**Fig 6:**
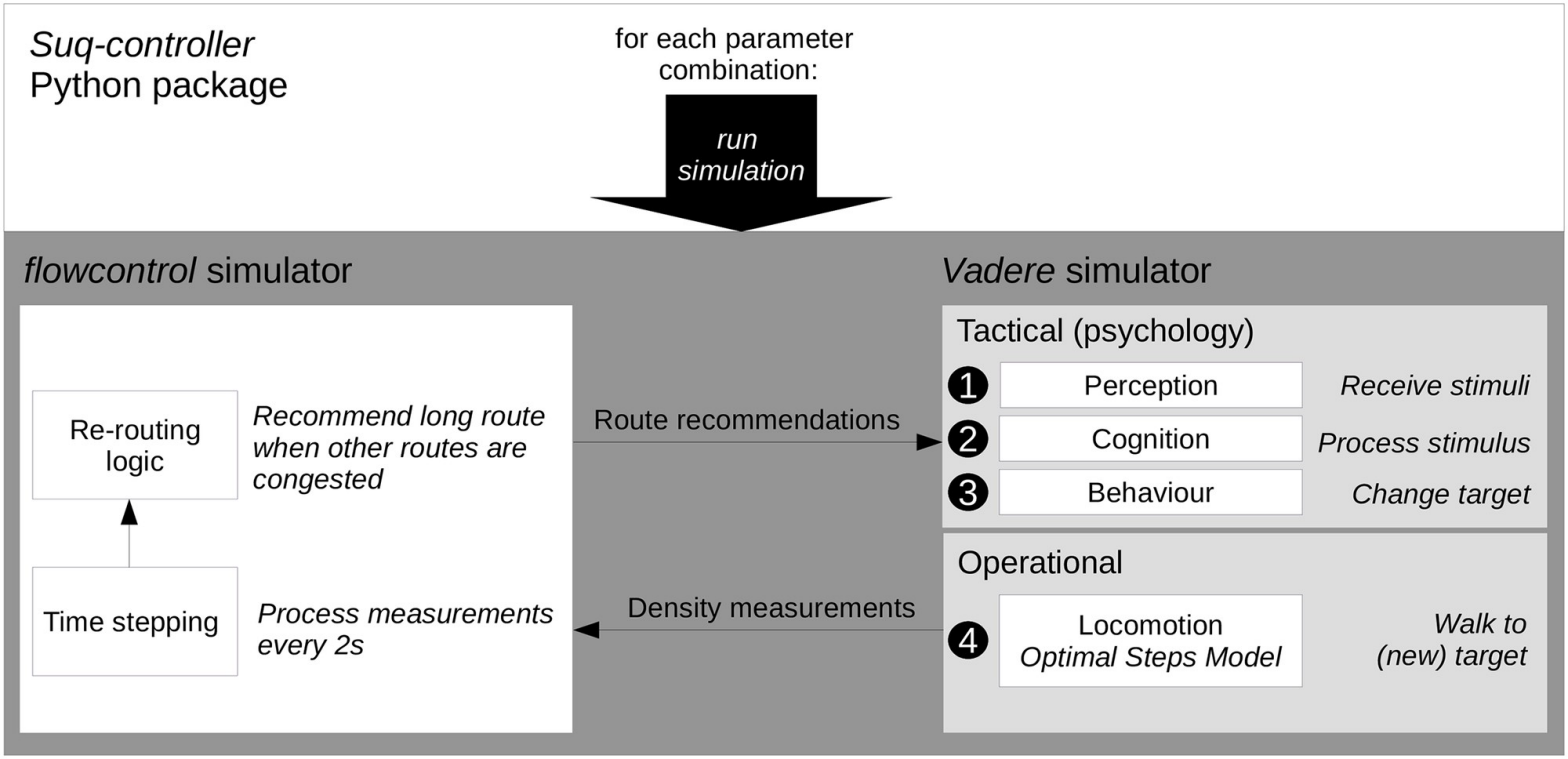
Overview of simulators and models that we use for the traffic simulation of the Münchner Freiheit scenario (see Fig 5). We test 18 (= 2+2x8) parameter combinations: for each of the two groups, we simulate the crowd flow prior to information and for the 8 message designs. Every 2s, the re-routing logic receives density measurements from the crowd simulation. If the long route is less congested than the others, a route recommendation is sent to the crowd model (*Vadere* simulator). The route recommendation is perceived, processed and finally changes the route distribution. Therefore the mobility behavior (Optimal Steps Model) changes.

We implemented the models in the *Crownet* simulation framework. *Crownet* can be used to simulate the redirection of crowds using mobile communication. In this study we leave out the mobile communication and look at the redirection and the locomotion behavior only, assuming that messages are delivered without fail. Please find the repository under github.com/roVer-HM/crownet/tree/route_choice_survey for more information. *Crownet* compromises the pedestrian dynamics simulator *Vadere* and the Python framework *flowcontrol*. *Vadere* is an open-source simulator that allows to simulate the locomotion and behavioral changes of a crowd. Please see vadere.org for more information. We implemented the re-routing logic in *flowcontrol*, see Fig 6. We implemented the route choice behavior in the psychology layer of *Vadere*. Every two seconds, density measurements from the crowd simulation (*Vadere*) are passed to the re-routing logic (*flowcontrol*). Based on this, the algorithm generates a route recommendation which is then passed back to the crowd model. For more details about the interaction of the simulators, we refer to [30]. We used the Python framework *suq-controller* to run parameter studies.

### Design of experiment

We conducted a simulation study that consists of 18 sub-experiments to portray the crowd flow of football fans, and of students and faculty associates. Each simulated sub-experiment reflects a condition from the survey. Two of the sub-experiments reflect the control condition for each group where we obtained participants’ route choice prior to them receiving any route information (see Fig 4 and S2F Table). The other 16 sub-experiments replicate the experimental conditions from the survey where different types of route information were provided (8 message designs for the students and faculty associates and 8 message designs for the football fans). We capture the flow of the crowd in response to the 8 types of message designs, based on the route distributions from Table 6. For each sub-experiment, we repeated the simulation five times to capture the variations between simulation runs due to stochastic effects. Note that stochasticity is used to model the variations in real crowds, e.g. a normally distributed walking speed is modeled by drawing the agents’ desired walking speeds from a random distribution.

In each experiment, we measured the crowd density *d* [33] every 0.4*s*. It is defined as *d* = *N*/*A*, where *N* is the number of pedestrians and *A* is the area of the measurement area. In our scenario, the three measurement areas correspond to the three routes, see Fig 5. We expected high densities in the reference configurations, since about half of the virtual persons take the short route.

**Fig 7:**
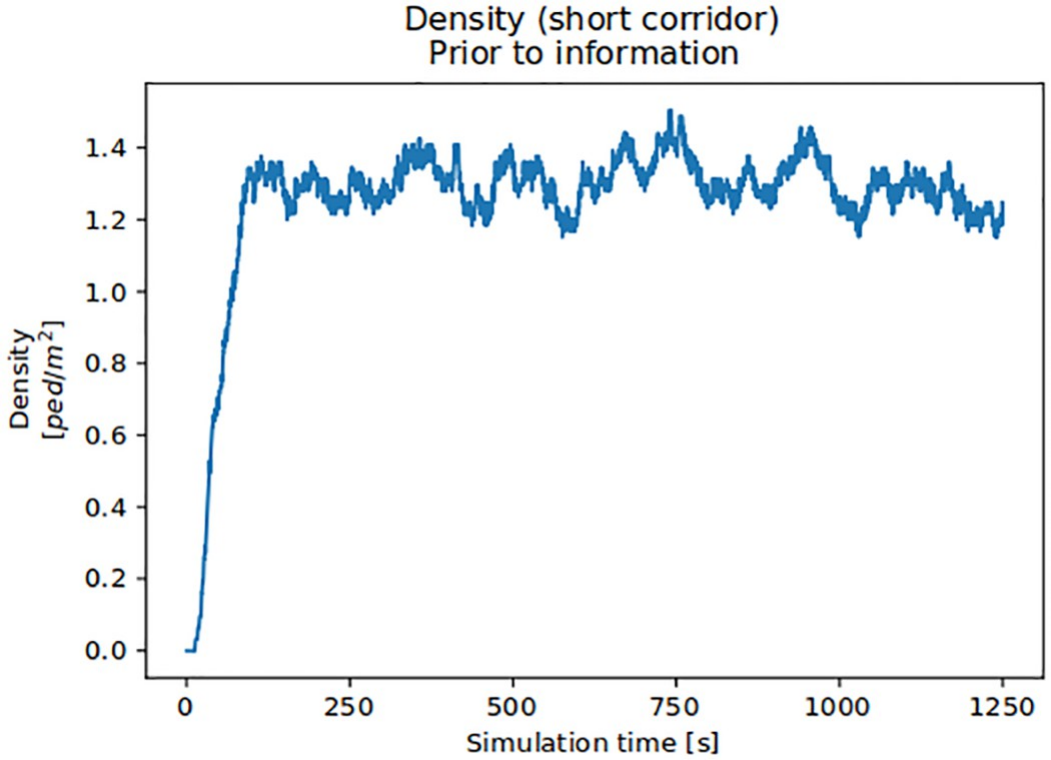
Crowd density over time along the short route for the fans prior to information. A steady state is reached after 250*s*. In this study, we looked at the steady state only, thus, neglecting any measurement data taken < 250*s*.

Regardless of the configuration, we observed that after about 250*s* of a ramp-up period, the system reaches a steady state, that is, density values oscillated about a stable mean, see Fig 7. Thus, we removed all density values before 250*s* and only evaluated the remaining 1000*s* to get reliable measurements that can be compared.

### Simulation results

**Fig 8:**
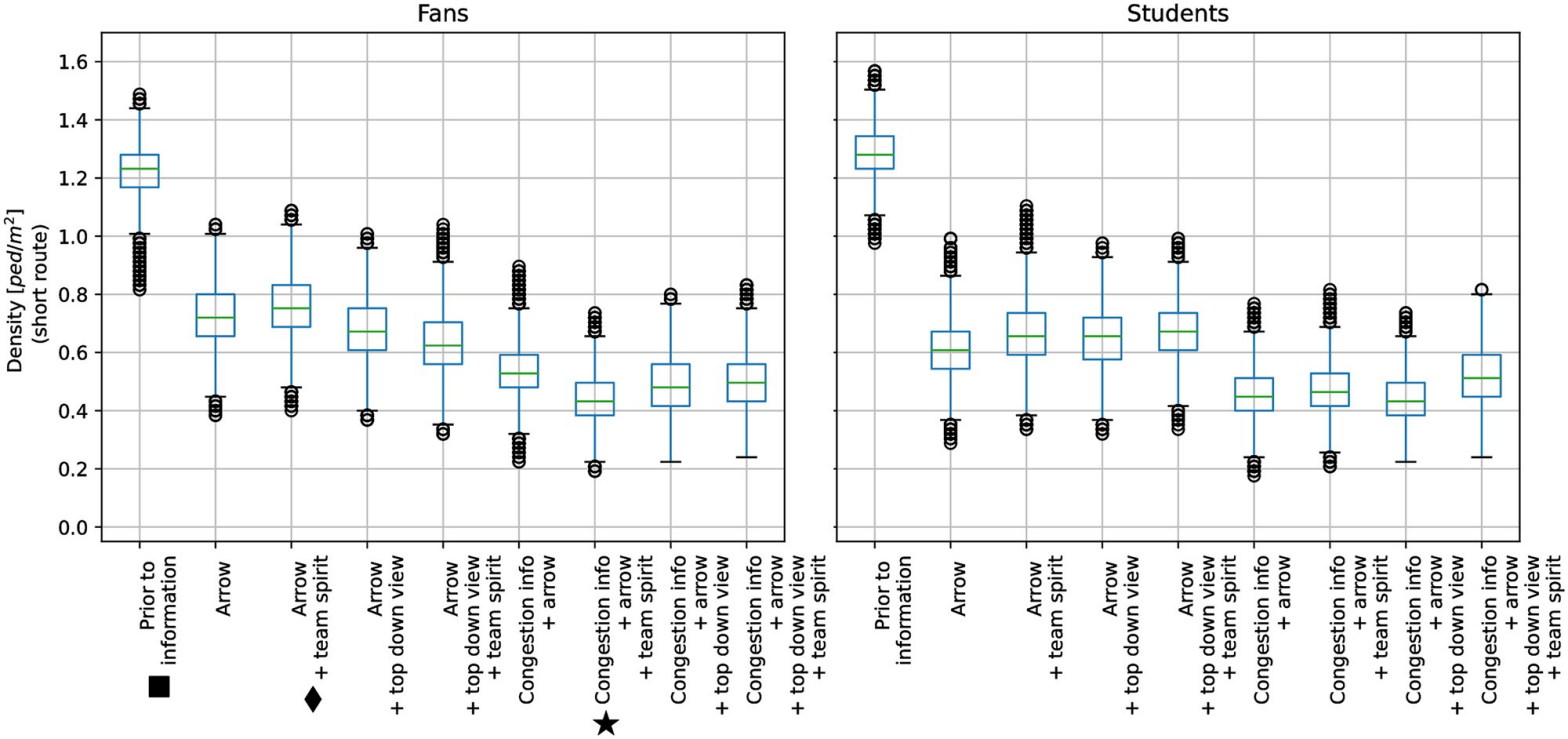
Boxplots of densities along the short route for the different message designs. The 25% and 75%-quartiles are represented by the boxes. The thick black line in the box represents the median value. Whiskers do not extend up and down from the box more than 1.5 times the interquartile range (75%-quartile—25%-quartile). Values outside the whiskers are considered as outliers (gray dots). Prior to information, the densities were the highest: the medians were above 1.2*ped*/*m*^2^ (fans: ▪) and some outliers were even above 1.5*ped*/*m*^2^. When a route recommendation was provided, the congestion was alleviated: the densities were lower compared to the setting prior to information. If the message depicts *congestion information* (boxplots 6–9), the densities were smaller than for message designs in which this component was missing (boxplots 2–5). We observed the largest difference for the fans when we provided *congestion information* in addition to the *arrow* and *team spirit* (compare ♦ and ★).

**Fig 9:**
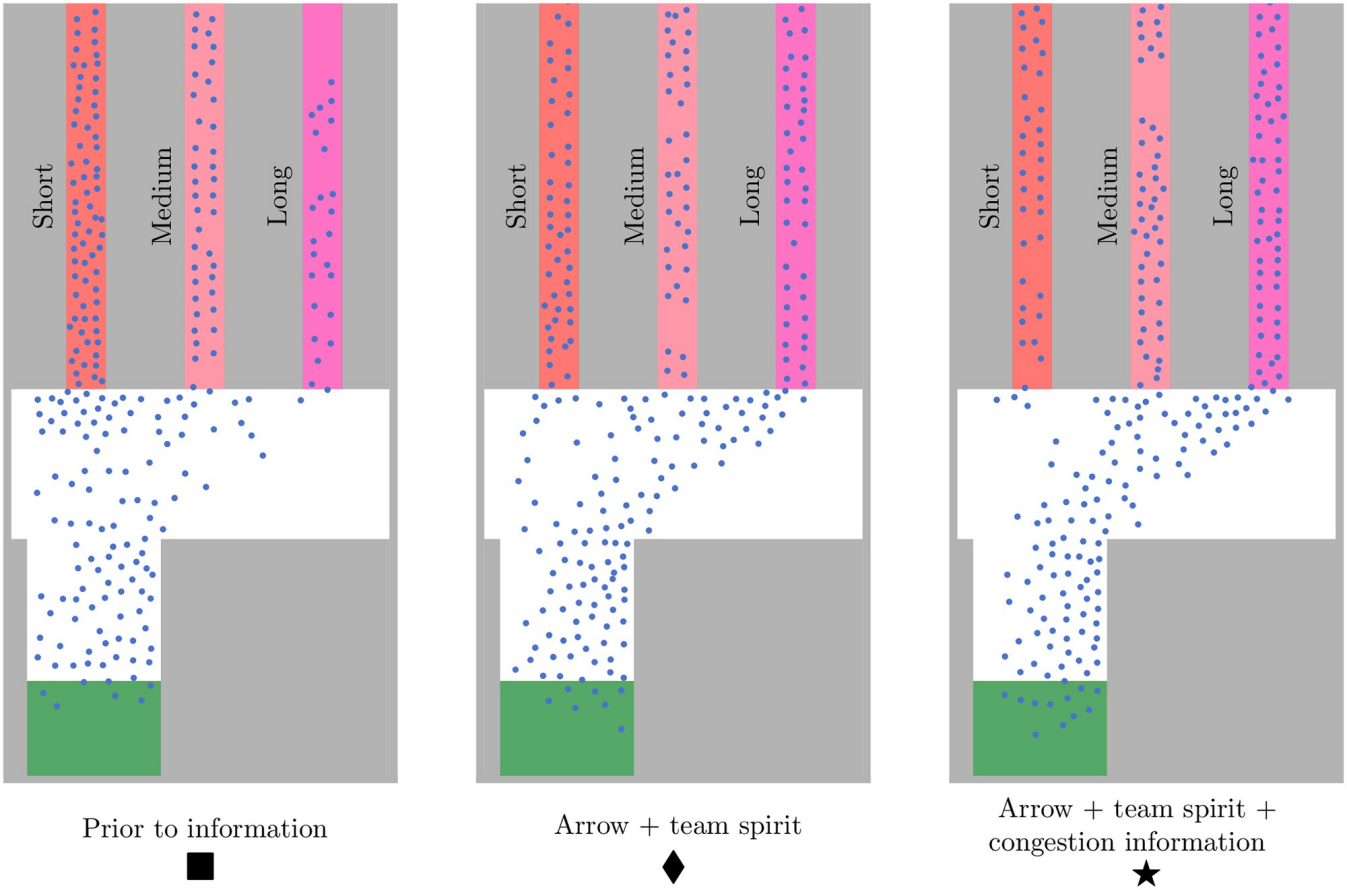
Screenshot of a simulation scenario with virtual fans to showcase the impact of route recommendations on pedestrian traffic. The virtual pedestrians distribute among the routes according to probabilities derived from the route preferences that football fans reported in the survey. The snapshot is taken after 300*s*. Prior to receiving route suggestions, most football fans take the short route which leads to higher densities along that route (▪). With the guidance system switched on, the virtual fans receive a message recommending the long route when the other routes are traveled more frequently. The message designs for screenshots ♦ and ★ include the components *arrow* pointing to the long route and appeal to the *team spirit*. In ★, information on the congestion situation is added. Both designs resolve the congestion in the short route, but congestion information (★) makes even more people choose the long route.

Prior to the agents receiving route information, we observed congestion in front of and along the short route, that is, the densities for both groups were sufficiently high to jam the bottlenecks: the medians were above 1.2*ped*/*m*^2^, and some outliers were even above 1.5*ped*/*m*^2^, see Fig 8. We could also observe this in the simulation screenshots, see Fig 9 (▪). The short route was congested, while the medium and long route were not. In fact the long route was almost empty. The congestion was alleviated once route recommendations were provided: we observed that the median densities dropped below 0.8*ped*/*m*^2^. Only outliers, density values larger than *Q*3 + 1.5(*Q*3 − *Q*1) where *Q*1, *Q*3 are the 25%-,75%-quartiles, are still above 1.0*ped*/*m*^2^. The lowest median densities occurred for message designs that included *congestion information*. This is in line with the higher compliance with suggestions reported in the survey when *congestion information* was provided and which our model translates to lower percentages of virtual pedestrians on the short route. For example, when the virtual fans did not receive *congestion information*, the median density was about 0.75*ped*/*m*^2^ for the message design with an *arrow* and an appeal to *team spirit*, see Fig 8 (♦). With *congestion information*, the median density dropped to 0.45*ped*/*m*^2^, see Fig 8 (★). Again, this was visible in the simulation (Fig 9): both message designs (♦, ★) alleviated the congestion along the short route (▪). However, adding *congestion information* to the message design made even more people choose the long route: compare (♦, ★) in Fig 9 and see (S1 Video). The density situation remains unchanged in rare cases: the whiskers in the boxplots 2–9 overlap, see Fig 8. The overlap stems from the large spread of the density (see the span of the whiskers in Fig 8), which is about 0.5*ped*/*m*^2^ for all message designs. We also find that providing route recommendation does not always improve the density situation in our scenario. The boxplots of the unguided setting (’prior to information’) overlap with some boxplots with route recommendation, see Fig 8. However, adding *congestion information* to the message design always reduces the density for the students and faculty associates. No overlap remains in the boxplots 1 (’prior to information’) and 6–9 (message design including *congestion information*), see Fig 8 (right). The overlap vanishes for the fans only in one message design (★).

## Discussion

The goal of this study was to assess how the design of messages in a guidance application affects pedestrians’ route choice and congestion. For this we conducted a survey among two study groups, football fans as well as students and faculty associates, who were presented with different message designs to redirect them at the train station Münchner Freiheit.

### Findings

Importantly, the results of our online visualization study showed that a certain proportion of people are willing to be rerouted within train stations through app recommendations. When we translated the reported route preferences into probabilities for route choices, we saw that, in the absence of a route recommendation system, every second person would have taken the shortest route. Once rerouting information was provided, this dropped as low as one in four people taking the shortest route, depending on the message design.

Interestingly, the survey revealed that people are more likely to follow a route recommendation when it includes real-time congestion information. We find this very plausible, because it explains to the participants why they should detour, thus improving compliance. We conclude that justifications or explanations such as real-time congestion information should accompany the practical instructions on where to go. This supports Holly Carter et al.’s findings in [17, 18] who found that providing practical instructions combined with explanations for the required behaviour resulted in highest compliance. In fact, our study results extend this conclusion to normal traffic situations.

We also found that football fans can be more easily redirected if one appeals to their team spirit, i.e., their pre-existing social identity. We observed that adherence increased for the football fans when we added an appeal to support the team alongside an arrow depicting the path and the congestion information. Notably, in the presence of more components (i.e., additional information), the difference was no longer statistically significant. This could be either a statistical coincidence or an indication of the sensitivity of the social identity to environmental conditions. We believe the latter is more likely, as other studies [17, 18] have already demonstrated the impact of social identities on cooperation to instructions. Additionally, there are potential explanations for the non-significant outcome, such as the possibility of information overload, where the addition of yet another piece of information does not result in a different response, or that the person already had enough information to decide to alter their behavior. Another important caveat is that appeals to do something beneficial for the group do not have an effect if people do not identify as a member of that group or if social identification with that team is low. We see this in the student and faculty associates group where the appeal did not affect them and they had significantly lower levels of social identification with the fan community compared to the football fans.

Importantly, the combination of information provided had different effects on route choice and no one specific aspect of message design had the most effect. The interactions between the different message types make it difficult to identify the ideal message design. Fortunately, adding a component never led to a deterioration in adherence. We believe that one must to tailor the message design to the use case and that further research is needed to see which combinations are reliably successful.

We also conducted computer simulations to see which pedestrian flows ensue when one translates the preferences reported in the survey into actual route choices. Our simulation results demonstrated that a congestion on the short route that appeared when virtual agents were left to their ‘natural’ route choice could be alleviated with any one of the message designs. Densities became lower, especially when congestion information is provided which is consistent with our findings from the survey.

### Limitations and open issues

There remain some limitations and open issues which we would like to address.

We consider our simulation model as a proof of concept that can be successfully used to qualitatively investigate the effect of messages. If one desires to make quantitative statements about the density or flow then the model needs further refinement regarding the topography and parameter estimates such as the arrival rate.

The results on route choice behavior are scenario-specific. For a different topography where, for example, there are only two routes, one would conduct the survey and simulation with the new topography. However, our proposed methodology would remain the same.

In a real-world scenario, it seems unlikely that everybody uses and looks at an application when traffic managers want to provide them with route recommendations. We did not include this in our simulation. Follow-up studies should model this uncertainty in the percentage of persons that can reached via an app or, possibly, verbal exchange with other pedestrians.

Also, we assumed that individuals choose their route independently from other group members since the participants completed the experiment individually. In reality, in-group members might base their decision on others, because they want to stay together as a crowd. However, we believe that this helps rather than hinders the success of the rerouting, because in reality, subgroups are likely to form within the crowd. Hence, people who do not use an application could still be redirected by following their in-group members. It would be interesting to model this in the route assignment model.

Similarly, we assume that people are familiar with the train station Münchner Freiheit. In the survey, we realized this by providing participants with photographs and map views that depict, among other things, different route lengths. When people followed the recommendation to take the long route, we assumed that they were aware of the longer distance and consciously accepted that. We fear that, when people are less aware of the consequences of their choices they might lose trust in the app, once they realize that they were sent along a long path. This risk should be mitigated by communicating that, while the route is longer, it is safer or, if true, faster. Looking into choices of participants who are left unfamiliar with the environment would be an interesting follow-up study.

When analyzing the data, we realized that there are most likely additional confounding factors, such as demographic characteristics. However, we did not anticipate confounding factors and therefore made no provisions in our study design. Nevertheless, we believe that the demographic characteristics do not affect our results in a major way. We argue that the demographics of the overall populations are well represented in the survey with its high number of participants.

In our scenario, we used the density of people to evaluate which message designs successfully alleviate congestion. However, it highly depends on the scenario which criterion is suitable to assess the effect of message designs. It would be interesting to also consider the throughput or travel time in future studies.

## Conclusion

We presented a survey combined with a computer simulation of pedestrian streams to asses the effect of message design in a navigation app on route preferences and on actual traffic. Participants from two groups, football fans as well as students and faculty associates, were presented with guiding information of different designs that would direct them at train station Münchner Freiheit right before a football match. In real life, certain areas of the station tend to congest on these occasions. We translated the route preferences reported by the participants into a traffic assignment model to inform a computer model of a crowd.

Our findings from the survey indicate that people are willing, in principle, to follow route recommendations provided by an app. However, there was no ideal message design to convince any kind of social group to change their behavior. Messages that appeal to the team spirit increased the compliance of football fans when combined with other message components, but had no effect on students and faculty associates where social identification was lower. We conclude that social identities indeed play a role in message design and suggest further research to assess how message design and social identity interplay. Combining instructions with explanations, in our case, real-time information on congestion, and thus a reason why one should follow the instruction, proved most effective in fostering compliance.

Our simulations studies showcase how reported behavioral changes translate into changes in crowd flow which resolved congestion in our application scenario.

By informing the computer model with survey data we presented a trans-disciplinary methodology to gain a holistic view on transportation systems. We believe that our novel methodology can be transferred to other application use cases, and thus, make decision-making more reliable.

## Supporting information

S1 Appendix Would i walk according to a route recommendation?. (PDF)

S2 Appendix Overview of statistical tests. (PDF)

S1 Table Survey: Raw data. (XLSX)

S2 Table Survey: Additional statistical analysis. (PDF)

S1 Video Traffic simulation. (MP4)
