# Supplementary material for: Designing mobile application messages to impact route choice: A survey and simulation study
Source: PLoS One. 2023 Apr 20;18(4):e0284540. doi: 10.1371/journal.pone.0284540 (PMC10118099; doi:10.1371/journal.pone.0284540)
Supplement: S1 Appendix — (PDF) [file pone.0284540.s001.pdf]

Would I walk according to a route recommendation?

Goal of this study

As part of the **roVer (https://www.hm.edu/allgemein/forschung_entwicklung/forschungsprojekte/projektdetails/wischhof/wischhof_koester_rover.de.html)** research project, the University of Applied Sciences Munich is conducting a survey to find out which route football fans take at an underground station to get to the train. This should make travelling safer and more comfortable.

How long does the survey take?

About 7 minutes.

What will I have to do if I take part in the survey?

You imagine that you are at a tube station to get on the train to a football match.

In the first step, you are given a view of the surrounding area.

In the next step, you get randomly assigned information about the environment.

This information would be communicated in reality using an app.

After the study is completed, the anonymised data is statistically analysed to understand influences on the choice of route.

Can I repeat the survey?

We ask that each person completes the survey only once.

Note

We recommend conducting the survey on a tablet or PC.

There are 23 questions in this survey.

Informed consent

By continuing with this survey, you are consenting to the following

1. I am at least 16 years old.
2. I have read and understood the introduction to the survey and what it entails.
3. My participation is voluntary, and I am free to stop the survey at any point.
4. Information collected will be analysed by the research team at the Hochschule München University of Applied Sciences to help understand pedestrians's reaction to route recommendations at an underground train station.
5. I understand that after the study will be made 'open data'. I understand that this means the anonymised data will be publicly available and may be used for purposes not related to this study, and it will not be possible to identify me from these data.

I agree with the points above.

*

Please choose **only one** of the following:

- ◯ Yes
- ◯ No

Introduction

Imagine that you and your family/friends are on your way to a football game at the *Allianz Arena* (soccer stadium in Munich) using public transport.

To get to the *Allianz Arena*, you have to change to the train at the Münchner Freiheit underground station, heading out of town.

To be informed about the current travel situation, you have an app installed.

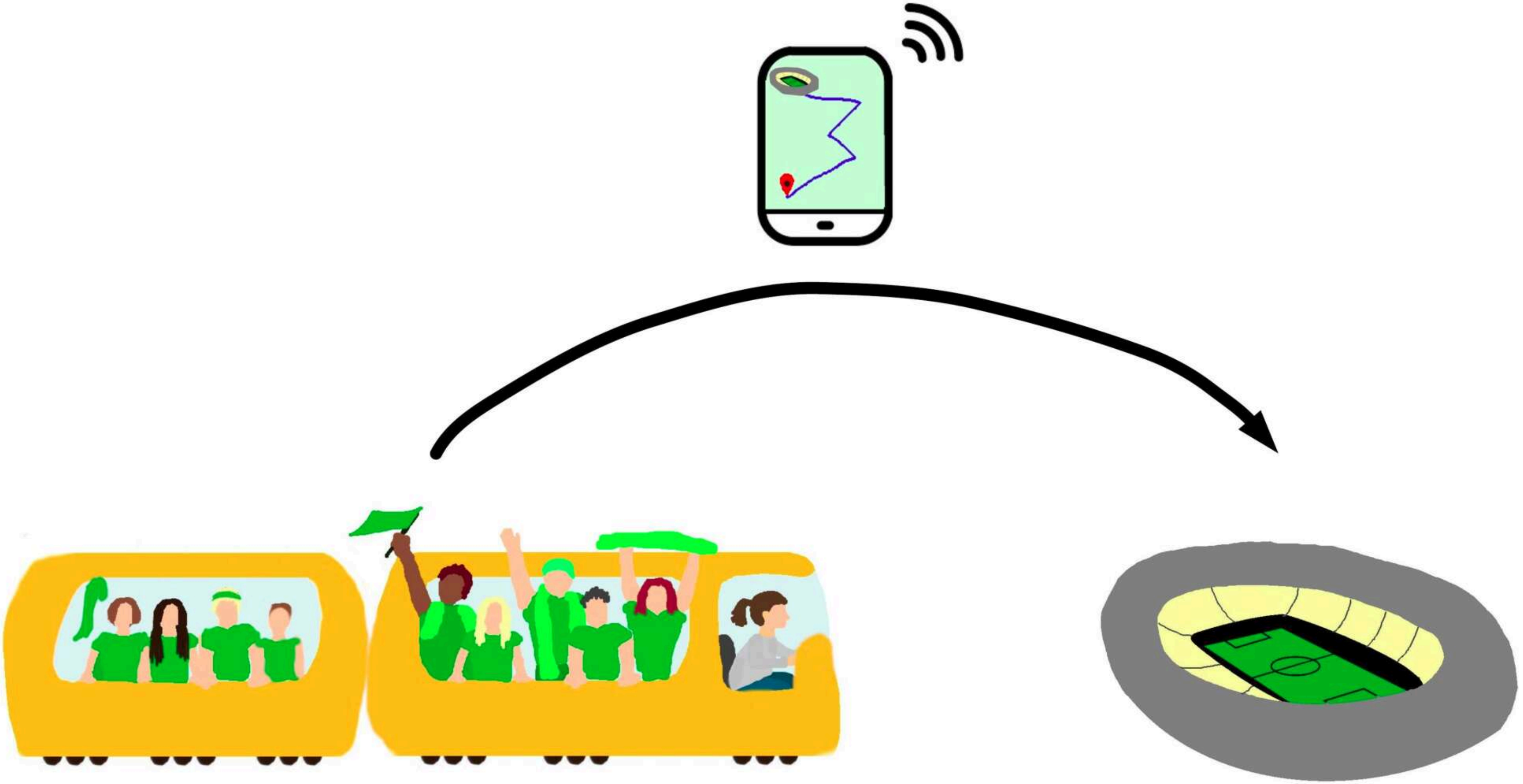

You are on your way to the underground station with your friends or family

You make predictions about the outcome of today's match and discuss the line-up. The atmosphere is good.

You arrive by bus at Münchner Freiheit and want to change to the train. As you do so, you see many other fans of your team. Everyone is looking forward to the game.

Please say how the following applies to you. *

Please choose the appropriate response for each item:

	Strongly disagree	Disagree	Neutral	Agree	Strongly agree
**I can imagine the situation well.**	◯	◯	◯	◯	◯

Being in a crowd of fans

Imagine you are at an underground station in the crowd of football fans.
What is important for you in this moment?
*

Please choose the appropriate response for each item:

	Strongly disagree	Disagree	Neutral	Agree	Strongly agree
**It is important for me to support my football team.**	○	○	○	○	○
**It is important for me to take care of my friends/family members at the train station.**	○	○	○	○	○
**It is important for me to support fans of my team.**	○	○	○	○	○
**It is important for me to travel safely.**	○	○	○	○	○
**It is important for me to walk together with my friends/family.**	○	○	○	○	○

Getting along with the enviroment

You want to get on the train.

There are two entrances to get to the train.

These entrances are marked as **blue U** in the top-down-view of the Münchner Freiheit. See below.

The black dot marks your current position.

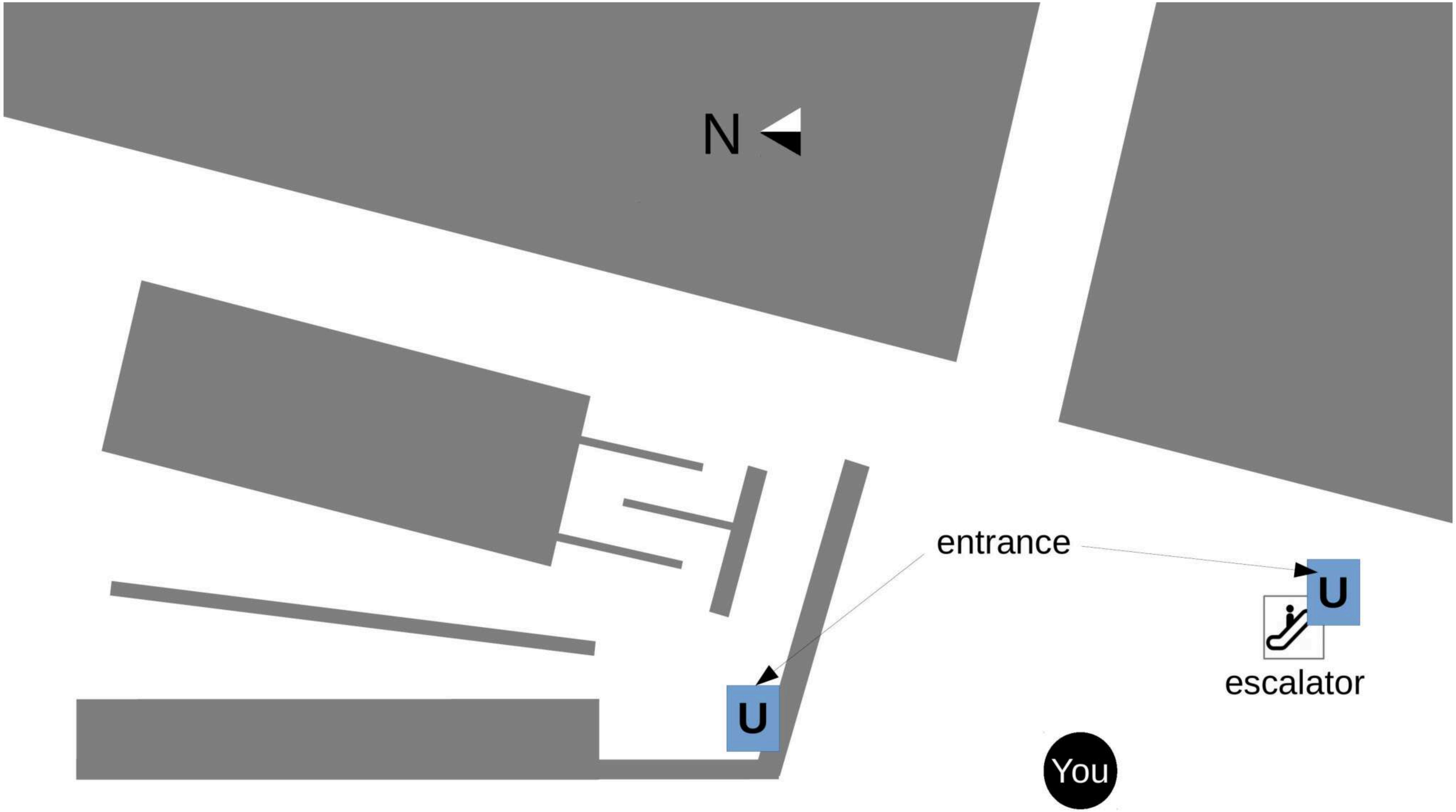

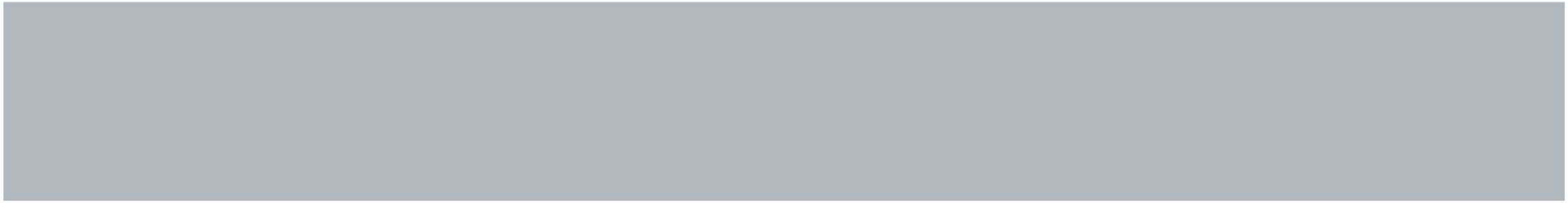

You look around and search for a way to the train.

When you look to the left, you see A.

B is right in front of you.

C is when you look to the right.

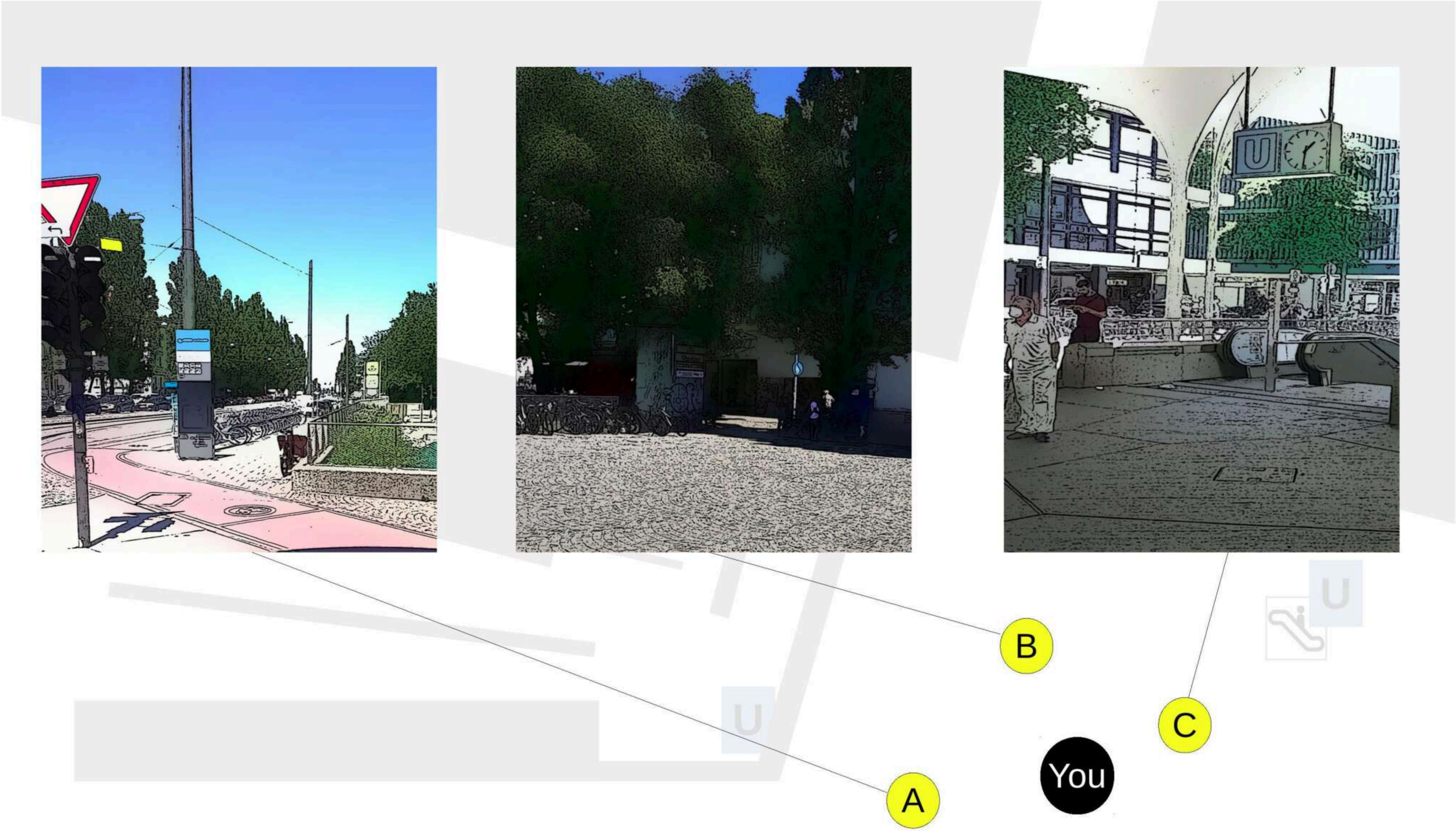

Which route would you choose?

How likely is it that you would choose the following routes? *

Please choose the appropriate response for each item:

	Very likely	Likely	Neutral	Unlikely	Very unlikely
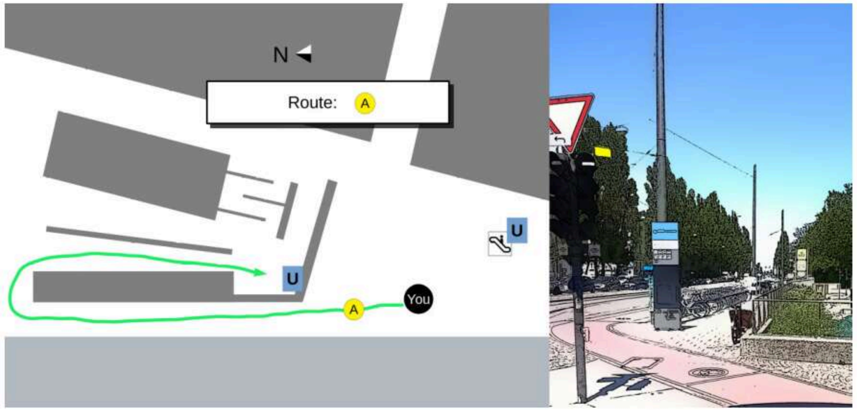	○	○	○	○	○
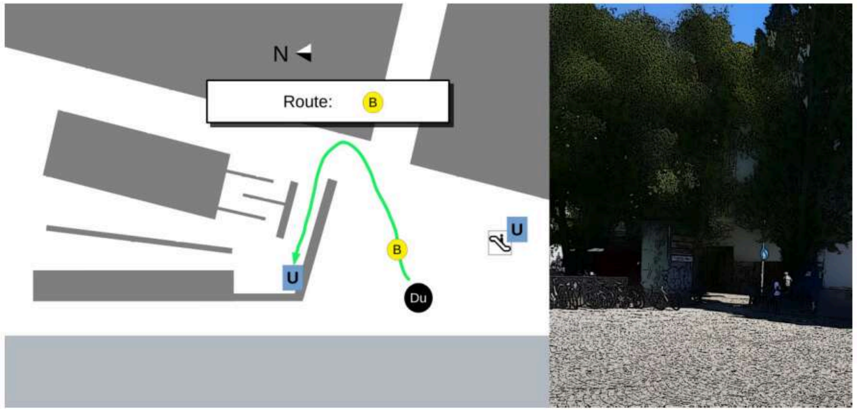	○	○	○	○	○

	Very likely	Likely	Neutral	Unlikely	Very unlikely
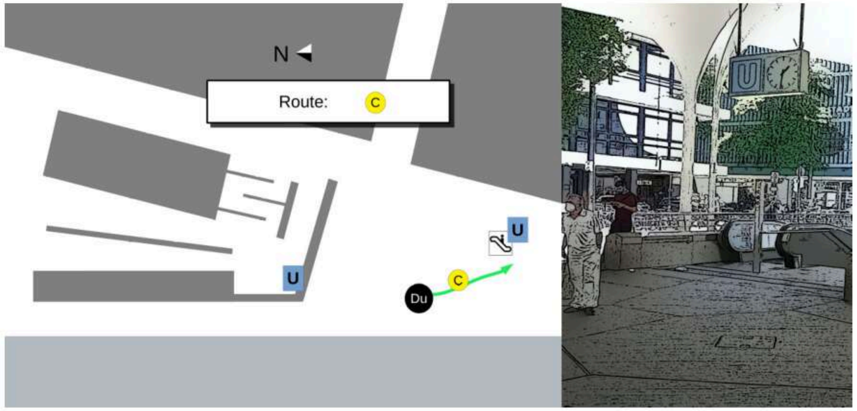	○	○	○	○	○

Please say how much the following points apply to you. *

Please choose the appropriate response for each item:

	Strongly agree	Agree	Neutral	Disagree	Strongly disagree
**I understand what the environment looks like.**	○	○	○	○	○
**I understand how to stay on the different routes.**	○	○	○	○	○
**I prefer short routes.**	○	○	○	○	○

Traffic app news

Suddenly you receive a notification from your traffic app.

{VarRandomCondition}

This field serves as check. It should display the condition that you picked from the drop down menu above (or the random assignment if this is activated)

To generate the final survey, replace "selectedCondition" by "randomCondition" and hide all dummy questions as well as the drop down menu.

{if(is_empty(drawRandomNumber), rand(1,9), drawRandomNumber)}

Draw a random number. Please ignore this field in the prototyping phase.

This is a dummy question for assigning conditions to subjects randomly.

It will not be visible in the final survey.

{if(is_empty(VarRandomCondition),if(drawRandomNumber<5,sprintf("B%s",drawRandomNumber),sprintf("A%s",drawRa[illegible]
5)),VarRandomCondition)}

Please ignore this field in the prototyping phase.

This is a dummy question for assigning conditions to subjects randomly.

It will not be visible in the final survey.

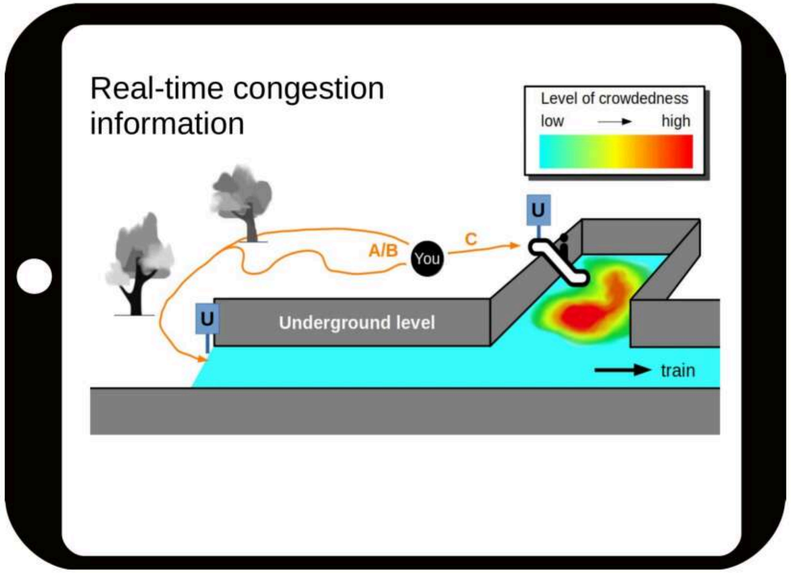

Only answer this question if the following conditions are met:

condition (/limesurvey/index.php/admin/questions/sa/view/surveyid/4/gid/2057/qid/27889) == "A0"

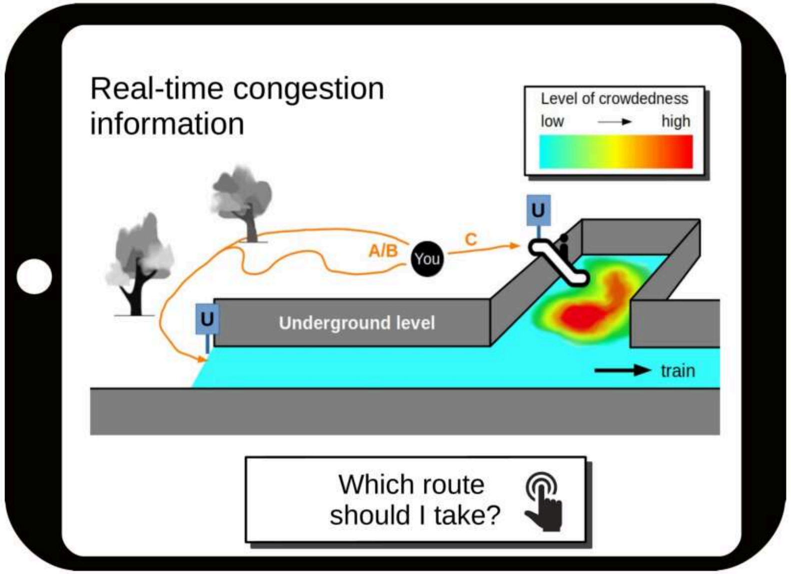

Only answer this question if the following conditions are met:

condition (/limesurvey/index.php/admin/questions/sa/view/surveyid/4/gid/2057/qid/27889) == "A1" or condition (/limesurvey/index.php/admin/questions/sa/view/surveyid/4/gid/2057/qid/27889) == "A2" or condition (/limesurvey/index.php/admin/questions/sa/view/surveyid/4/gid/2057/qid/27889) == "A3" or condition (/limesurvey/index.php/admin/questions/sa/view/surveyid/4/gid/2057/qid/27889) == "A4"

Please **use this route**
to avoid congested areas.

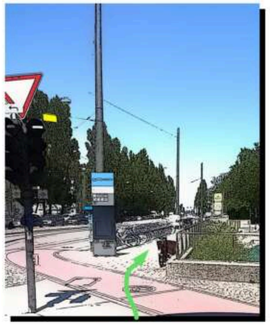

Only answer this question if the following conditions are met:

condition (/limesurvey/index.php/admin/questions/sa/view/surveyid/4/gid/2057/qid/27889) == "A1" or condition (/limesurvey/index.php/admin/questions/sa/view/surveyid/4/gid/2057/qid/27889) == "B1"

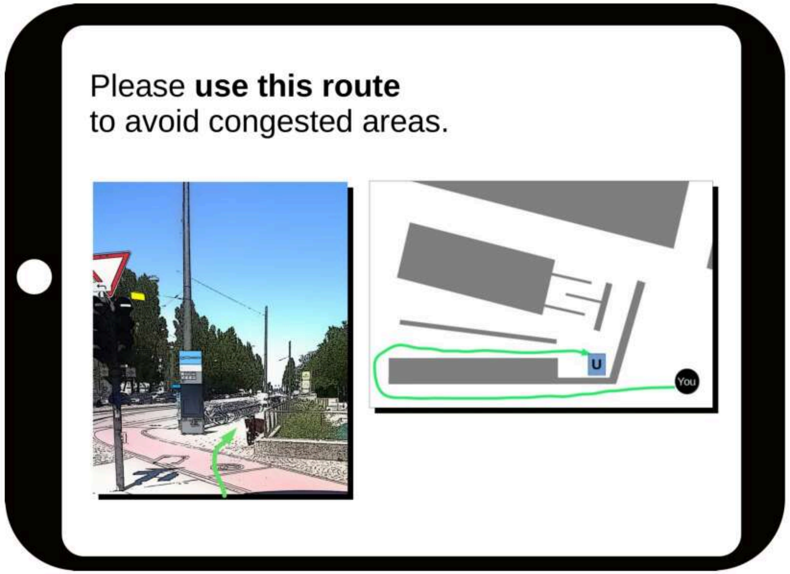

Only answer this question if the following conditions are met:

condition (/limesurvey/index.php/admin/questions/sa/view/surveyid/4/gid/2057/qid/27889) == "A2" or condition (/limesurvey/index.php/admin/questions/sa/view/surveyid/4/gid/2057/qid/27889) == "B2"

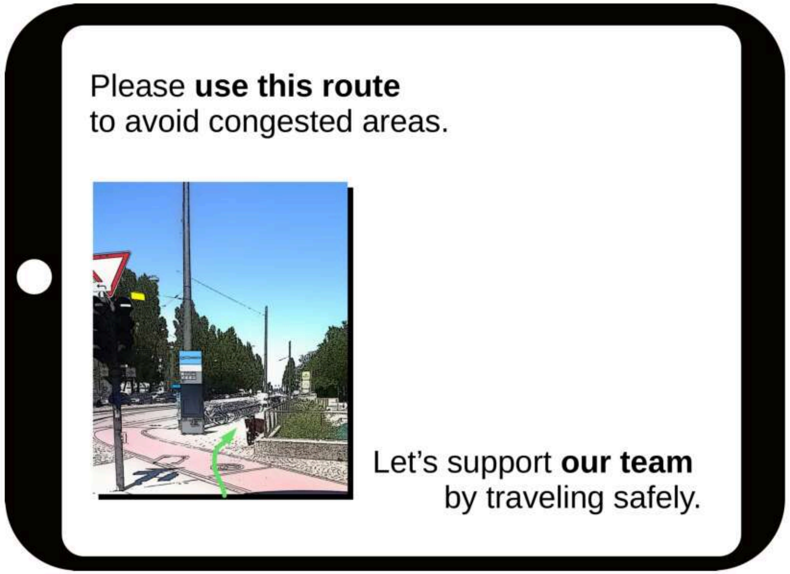

Only answer this question if the following conditions are met:

condition (/limesurvey/index.php/admin/questions/sa/view/surveyid/4/gid/2057/qid/27889) == "A3" or condition (/limesurvey/index.php/admin/questions/sa/view/surveyid/4/gid/2057/qid/27889) == "B3"

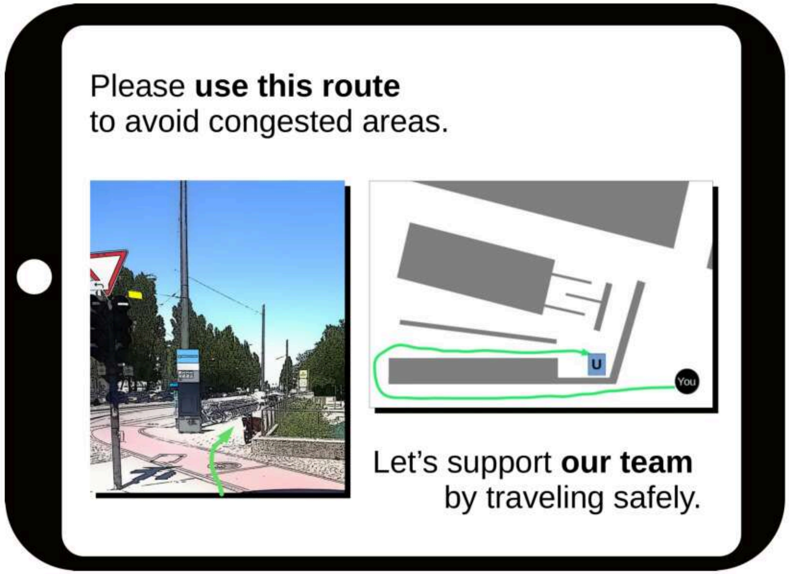

Only answer this question if the following conditions are met:

condition (/limesurvey/index.php/admin/questions/sa/view/surveyid/4/gid/2057/qid/27889) == "A4" or condition (/limesurvey/index.php/admin/questions/sa/view/surveyid/4/gid/2057/qid/27889) == "B4"

How likely is it that you choose the following routes?

*

Please choose the appropriate response for each item:

	Very likely	Likely	Neutral	Unlikely	Very unlikely
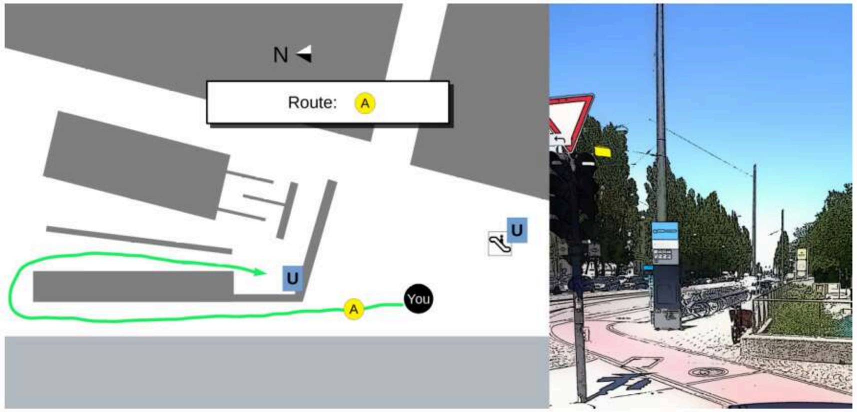	◯	◯	◯	◯	◯
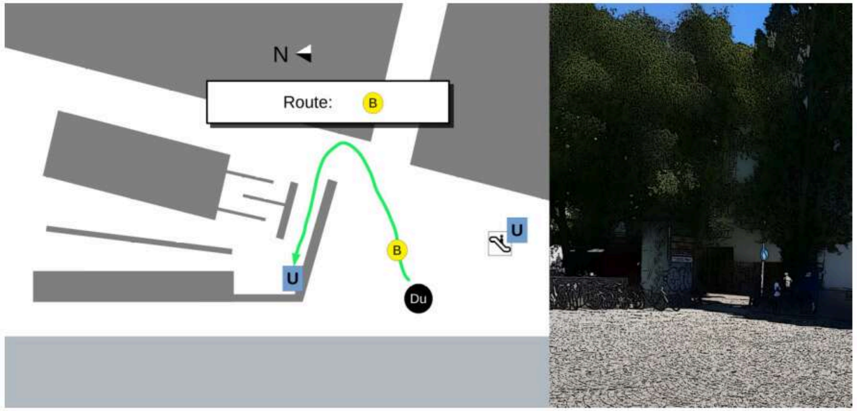	◯	◯	◯	◯	◯

	Very likely	Likely	Neutral	Unlikely	Very unlikely
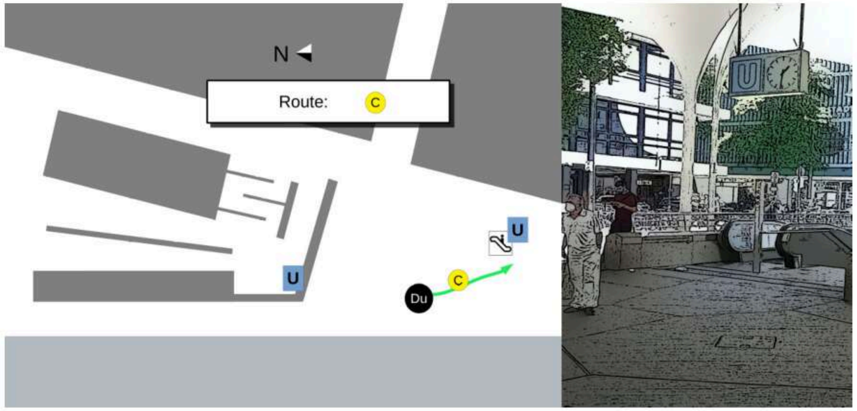	○	○	○	○	○

Please indicate how well the following statements apply to you. *

Please choose the appropriate response for each item:

	Strongly disagree	Disagree	Neutral	Agree	Strongly agree
**I need more information.**	◯	◯	◯	◯	◯
**I need less information.**	◯	◯	◯	◯	◯
**The information is engaging to me.**	◯	◯	◯	◯	◯
**I can understand the information quickly.**	◯	◯	◯	◯	◯
**I would follow the route recommendation only if still catch my train.**	◯	◯	◯	◯	◯
**The marked route helps me to decide where to go.**	◯	◯	◯	◯	◯
**The coloured map showing how busy it is helps me decide where to go.**	◯	◯	◯	◯	◯
**The photograph(s) help(s) me to decide where to go.**	◯	◯	◯	◯	◯

What do you think about the navigation app?

Please rate how well the statements apply to you. *

Please choose the appropriate response for each item:

	Strongly disagree	Disagree	Neutral	Agree	Strongly agree
**I think the traffic app works reliably.**	◯	◯	◯	◯	◯
**I think that the recommendations are fair for all.**	◯	◯	◯	◯	◯
**I feel motivated to follow the route recommendation.**	◯	◯	◯	◯	◯
**I think the displayed information helps me to travel more safely.**	◯	◯	◯	◯	◯
**I think that I can reduce congestion if I follow the recommendation.**	◯	◯	◯	◯	◯
**I can imagine being part of the fan community.**	◯	◯	◯	◯	◯

General data

How old are you? *

Choose one of the following answers

Please choose **only one** of the following:

- ◯ <18
- ◯ 18-25
- ◯ 26-35
- ◯ 36-50
- ◯ 51-65
- ◯ >65

To which gender identity do you most identify? *

Choose one of the following answers

Please choose **only one** of the following:

- ◯ Female
- ◯ Male
- ◯ Prefer not to answer
- ◯ See my comment (please provide a comment)

Make a comment on your choice here:

What device did you use to take the survey? *

❗ Choose one of the following answers
Please choose **only one** of the following:

- ◯ Smart phone/mobile phone
- ◯ Personal computer/laptop
- ◯ Tablet
- ◯ Device not listed here

Make a comment on your choice here:

Thank your for participating.

With your answers you help us to make local transport safer.
We would be happy if you forwarded the link to the survey to other people.

You can find out more about us and the research project **here (https://www.hm.edu/allgemein/forschung_entwicklung/forschungsprojekte/projektdetails/wischhof/wischhof_koester_rover.de.html)**.

29.10.2022 – 14:48

Submit your survey.
Thank you for completing this survey.
