# Supplementary material for: Designing mobile application messages to impact route choice: A survey and simulation study
Source: PLoS One. 2023 Apr 20;18(4):e0284540. doi: 10.1371/journal.pone.0284540 (PMC10118099; doi:10.1371/journal.pone.0284540)
Supplement: S2 Appendix — (PDF) [file pone.0284540.s002.pdf]

Overview of statistical tests

1 Mann-Whitney U test

The ‘Mann-Whitney U test’, also known as the ‘two-sample Wilcoxon test’ or ‘Wilcoxon rank-sum test’ is a non-parametric statistical that is used to test for a difference between two independent samples [1,2,3,4]. It is based on rank sums and it is used instead of the t-test when the data is not normally distributed [1]:

- Null hypothesis: There is no difference (in terms of central tendency) between the two samples.
- Alternative hypothesis: There is a difference (with respect to the central tendency) between the samples.

In our study, we perform multiple Mann-Whitney U tests for which we report the Wilcoxon rank sum statistic W and the corresponding p-Value. We used the following implementation:

Programming language	Package	function	Note
R	stats 3.6.2	`wilcox.test(x,y, paired=FALSE, ...)`	We set ‘paired=FALSE’ to carry out a Wilcoxon rank sum test (equivalent to the Mann-Whitney test)

For more details, please find the R documentation:
https://www.rdocumentation.org/packages/stats/versions/3.6.2/topics/wilcox.test (21.02.2023)

2 Kruskal-Wallis test

The ‘Kruskal-Wallis test,’ also known as the ‘Kruskal-Wallis rank sum test’ or ‘one-way ANOVA on ranks’ is an extension of the Mann-Whitney U test for comparing two or more independent samples [1,3]. It is used instead of the one-way analysis of variance (ANOVA) when the data is not normally distributed [1]:

- Null hypothesis: The independent samples all have the same central tendency and therefore come from the same population.
- Alternative hypothesis: At least one of the independent samples does not have the same central tendency as the others and therefore comes from a different population.

In our study, we perform multiple Kruskal-Wallis tests for which we report the statistic H and the corresponding p-Value. We used the following implementation:

Programming language	Package	function	Note
R	stats 3.6.2	`kruskal.test(x, …)`	Equivalent to the function wilcox.test in case of two samples

For more details, please find the R documentation:
https://www.rdocumentation.org/packages/stats/versions/3.6.2/topics/kruskal.test (21.02.2023)

References

[1] DATAtab: DATAtab Team (2023). DATAtab: Online Statistics Calculator. DATAtab e.U. Graz, Austria. https://datatab.net/ (21.02.2023)

[2] David F. Bauer (1972). Constructing confidence sets using rank statistics. *Journal of the American Statistical Association* **67**, 687--690. 10.1080/01621459.1972.10481279.

[3] Myles Hollander and Douglas A. Wolfe (1973). *Nonparametric Statistical Methods*. New York: John Wiley & Sons.

[4] Mann, H. B., & Whitney, D. R. (1947). On a test of whether one of two random variables is stochastically larger than the other. The annals of mathematical statistics, 50-60.
