# Supplementary material for: Designing mobile application messages to impact route choice: A survey and simulation study
Source: PLoS One. 2023 Apr 20;18(4):e0284540. doi: 10.1371/journal.pone.0284540 (PMC10118099; doi:10.1371/journal.pone.0284540)
Supplement: S2 Table — (PDF) [file pone.0284540.s004.pdf]

Tab. S2A. Effect of message component on how students & faculty associates evaluate the route attractiveness (Mann Whitney U test). If adding a component makes a significant difference ($p < 0.05$) we marked the corresponding p-Value bold. The mean values '(mean = ...)' represent the respective mean values of the route attractivenesses (see Tab. S2H for the full statistics of the route attractivenesses).

Students & faculty associates

Route	Added component	Message design 1	Message design 2	p	W
Long	Congestion info	Arrow (mean = 3.787)	Congestion info + arrow (mean = 3.862)	0.7592	1317.5
Long	Congestion info	Arrow + top down view (mean = 3.938)	Congestion info + arrow + top down view (mean = 4.022)	0.8557	1434.0
Long	Congestion info	Arrow + team spirit (mean = 3.696)	Congestion info + arrow + team spirit (mean = 3.700)	0.8356	1368.0
Long	Congestion info	Arrow + top down view + team spirit (mean = 3.823)	Congestion info + arrow + top down view + team spirit (mean = 3.885)	0.3327	1708.5
Long	Team spirit	Congestion info + arrow (mean = 3.862)	Congestion info + arrow + team spirit (mean = 3.700)	0.6071	1530.0
Long	Team spirit	Congestion info + arrow + top down view (mean = 4.022)	Congestion info + arrow + top down view + team spirit (mean = 3.885)	0.9810	1376.5
Long	Team spirit	Arrow (mean = 3.787)	Arrow + team spirit (mean = 3.696)	0.6422	1383.5
Long	Team spirit	Arrow + top down view (mean = 3.938)	Arrow + top down view + team spirit (mean = 3.823)	0.3331	2205.5
Long	Top down view	Congestion info + arrow (mean = 3.862)	Congestion info + arrow + top down view (mean = 4.022)	0.5715	1224.5
Long	Top down view	Congestion info + arrow + team spirit (mean = 3.700)	Congestion info + arrow + top down view + team spirit (mean = 3.885)	0.3818	1384.5
Long	Top down view	Arrow (mean = 3.787)	Arrow + top down view (mean = 3.938)	0.4979	1418.5
Long	Top down view	Arrow + team spirit (mean = 3.696)	Arrow + top down view + team spirit (mean = 3.823)	0.6822	1663.0
Medium	Congestion info	Arrow (mean = 2.872)	Congestion info + arrow (mean = 3.483)	**0.0117**	989.0
Medium	Congestion info	Arrow + top down view (mean = 2.769)	Congestion info + arrow + top down view (mean = 3.622)	**0.0003**	884.5
Medium	Congestion info	Arrow + team spirit (mean = 2.946)	Congestion info + arrow + team spirit (mean = 3.620)	**0.0031**	949.5
Medium	Congestion info	Arrow + top down view + team spirit (mean = 2.903)	Congestion info + arrow + top down view + team spirit (mean = 3.246)	0.1243	1598.5
Medium	Team spirit	Congestion info + arrow (mean = 3.483)	Congestion info + arrow + team spirit (mean = 3.62)	0.4871	1341.0
Medium	Team spirit	Congestion info + arrow + top down view (mean = 3.622)	Congestion info + arrow + top down view + team spirit (mean = 3.246)	0.1283	1601.5
Medium	Team spirit	Arrow (mean = 2.872)	Arrow + team spirit (mean = 2.946)	0.6933	1259.5
Medium	Team spirit	Arrow + top down view (mean = 2.769)	Arrow + top down view + team spirit (mean = 2.903)	0.5484	1895.5
Medium	Top down view	Congestion info + arrow (mean = 3.483)	Congestion info + arrow + top down view (mean = 3.622)	0.6519	1240.0
Medium	Top down view	Congestion info + arrow + team spirit (mean = 3.62)	Congestion info + arrow + top down view + team spirit (mean = 3.246)	0.1017	1792.5
Medium	Top down view	Arrow (mean = 2.872)	Arrow + top down view (mean = 2.769)	0.6865	1593.0
Medium	Top down view	Arrow + team spirit (mean = 2.946)	Arrow + top down view + team spirit (mean = 2.903)	0.7897	1783.5
Short	Congestion info	Arrow (mean = 3.128)	Congestion info + arrow (mean = 2.483)	**0.0019**	1804.0
Short	Congestion info	Arrow + top down view (mean = 3.338)	Congestion info + arrow + top down view (mean = 2.533)	**0.0009**	1974.5
Short	Congestion info	Arrow + team spirit (mean = 3.321)	Congestion info + arrow + team spirit (mean = 2.54)	**0.0010**	1895.5
Short	Congestion info	Arrow + top down view + team spirit (mean = 3.403)	Congestion info + arrow + top down view + team spirit (mean = 2.689)	**0.0011**	2514.5
Short	Team spirit	Congestion info + arrow (mean = 2.483)	Congestion info + arrow + team spirit (mean = 2.54)	0.9363	1438.0
Short	Team spirit	Congestion info + arrow + top down view (mean = 2.533)	Congestion info + arrow + top down view + team spirit (mean = 2.689)	0.4671	1270.0
Short	Team spirit	Arrow (mean = 3.128)	Arrow + team spirit (mean = 3.321)	0.4109	1196.5
Short	Team spirit	Arrow + top down view (mean = 3.338)	Arrow + top down view + team spirit (mean = 3.403)	0.7408	1948.0
Short	Top down view	Congestion info + arrow (mean = 2.483)	Congestion info + arrow + top down view (mean = 2.533)	0.9657	1311.0
Short	Top down view	Congestion info + arrow + team spirit (mean = 2.54)	Congestion info + arrow + top down view + team spirit (mean = 2.689)	0.5119	1422.5
Short	Top down view	Arrow (mean = 3.128)	Arrow + top down view (mean = 3.338)	0.4113	1393.0
Short	Top down view	Arrow + team spirit (mean = 3.321)	Arrow + top down view + team spirit (mean = 3.403)	0.6679	1658.5

Tab. S2B. Effect of message component on how fans evaluate the route attractiveness (Mann Whitney U test). If adding a component makes a significant difference ($p < 0.05$) we marked the corresponding p-Value bold. The mean values '(mean = ...)' represent the respective mean values of the route attractivenesses (see Tab. S2H for the full statistics of the route attractivenesses).

Fans

Route	Added component	Message design 1	Message design 2	p	W
Long	Congestion info	Arrow (mean = 3.427)	Congestion info + arrow (mean = 3.505)	0.6181	7099.5
Long	Congestion info	Arrow + top down view (mean = 3.55)	Congestion info + arrow + top down view (mean = 3.897)	**0.0331**	5433.0
Long	Congestion info	Arrow + team spirit (mean = 3.553)	Congestion info + arrow + team spirit (mean = 3.980)	**0.0047**	4107.0
Long	Congestion info	Arrow + top down view + team spirit (mean = 3.743)	Congestion info + arrow + top down view + team spirit (mean = 3.879)	0.6983	7076.0
Long	Team spirit	Congestion info + arrow (mean = 3.505)	Congestion info + arrow + team spirit (mean = 3.980)	**0.0069**	4157.0
Long	Team spirit	Congestion info + arrow + top down view (mean = 3.897)	Congestion info + arrow + top down view + team spirit (mean = 3.879)	0.6011	6443.0
Long	Team spirit	Arrow (mean = 3.427)	Arrow + team spirit (mean = 3.553)	0.5517	7048.5
Long	Team spirit	Arrow + top down view (mean = 3.55)	Arrow + top down view + team spirit (mean = 3.743)	0.1957	6856.0
Long	Top down view	Congestion info + arrow (mean = 3.505)	Congestion info + arrow + top down view (mean = 3.897)	**0.0305**	5006.0
Long	Top down view	Congestion info + arrow + team spirit (mean = 3.980)	Congestion info + arrow + top down view + team spirit (mean = 3.879)	0.1645	6028.0
Long	Top down view	Arrow (mean = 3.427)	Arrow + top down view (mean = 3.55)	0.5125	7570.5
Long	Top down view	Arrow + team spirit (mean = 3.553)	Arrow + top down view + team spirit (mean = 3.743)	0.1652	6299.0
Medium	Congestion info	Arrow (mean = 3.266)	Congestion info + arrow (mean = 3.534)	0.0731	6423.5
Medium	Congestion info	Arrow + top down view (mean = 3.207)	Congestion info + arrow + top down view (mean = 3.655)	**0.0021**	5006.5
Medium	Congestion info	Arrow + team spirit (mean = 3.078)	Congestion info + arrow + team spirit (mean = 3.539)	**0.0038**	4087.5
Medium	Congestion info	Arrow + top down view + team spirit (mean = 3.066)	Congestion info + arrow + top down view + team spirit (mean = 3.542)	**0.0022**	5678.0
Medium	Team spirit	Congestion info + arrow (mean = 3.534)	Congestion info + arrow + team spirit (mean = 3.539)	0.8731	5188.5
Medium	Team spirit	Congestion info + arrow + top down view (mean = 3.655)	Congestion info + arrow + top down view + team spirit (mean = 3.542)	0.4670	6535.0
Medium	Team spirit	Arrow (mean = 3.266)	Arrow + team spirit (mean = 3.078)	0.1820	8065.5
Medium	Team spirit	Arrow + top down view (mean = 3.207)	Arrow + top down view + team spirit (mean = 3.066)	0.3322	8066.0
Medium	Top down view	Congestion info + arrow (mean = 3.534)	Congestion info + arrow + top down view (mean = 3.655)	0.4556	5644.5
Medium	Top down view	Congestion info + arrow + team spirit (mean = 3.539)	Congestion info + arrow + top down view + team spirit (mean = 3.542)	0.9011	5508.5
Medium	Top down view	Arrow (mean = 3.266)	Arrow + top down view (mean = 3.207)	0.6396	8195.5
Medium	Top down view	Arrow + team spirit (mean = 3.078)	Arrow + top down view + team spirit (mean = 3.066)	0.9352	7045.5
Short	Congestion info	Arrow (mean = 3.643)	Congestion info + arrow (mean = 2.806)	**0.0000**	9855.0
Short	Congestion info	Arrow + top down view (mean = 3.414)	Congestion info + arrow + top down view (mean = 2.784)	**0.0003**	8143.0
Short	Congestion info	Arrow + team spirit (mean = 3.786)	Congestion info + arrow + team spirit (mean = 2.471)	**0.0000**	7940.5
Short	Congestion info	Arrow + top down view + team spirit (mean = 3.265)	Congestion info + arrow + top down view + team spirit (mean = 2.757)	**0.0023**	8857.5
Short	Team spirit	Congestion info + arrow (mean = 2.806)	Congestion info + arrow + team spirit (mean = 2.471)	0.0634	5993.5
Short	Team spirit	Congestion info + arrow + top down view (mean = 2.784)	Congestion info + arrow + top down view + team spirit (mean = 2.757)	0.9759	6192.0
Short	Team spirit	Arrow (mean = 3.643)	Arrow + team spirit (mean = 3.786)	0.3306	6851.5
Short	Team spirit	Arrow + top down view (mean = 3.414)	Arrow + top down view + team spirit (mean = 3.265)	0.3873	8015.0
Short	Top down view	Congestion info + arrow (mean = 2.806)	Congestion info + arrow + top down view (mean = 2.784)	0.8446	6060.5
Short	Top down view	Congestion info + arrow + team spirit (mean = 2.471)	Congestion info + arrow + top down view + team spirit (mean = 2.757)	0.0781	4740.5
Short	Top down view	Arrow (mean = 3.643)	Arrow + top down view (mean = 3.414)	0.1237	8799.5
Short	Top down view	Arrow + team spirit (mean = 3.786)	Arrow + top down view + team spirit (mean = 3.265)	**0.0024**	8555.5

Tab. S2C. Effect of information provision on how students & faculty associates evaluate the route attractiveness (Mann Whitney U test). If information provision makes a significant difference ($p < 0.05$) we marked the corresponding p-Value bold. Through information the long route becomes always more attractive (the means increase), while the short route always becomes less attractive (the means reduce). Also, the medium route becomes more attractive. The mean value always increases, but the difference is not always significant.

Students & faculty associates

Route	Condition	Route attractiveness (mean value)		p	W
		Prior to info.	*Info. provided*		
Long	Arrow + top down view	2.062	3.938	**0.0000**	3654.5
Long	Arrow + team spirit	2.196	3.696	**0.0000**	2542.5
Long	Congestion info + arrow	2.241	3.862	**0.0000**	2758.0
Long	Arrow + top down view + team spirit	2.145	3.823	**0.0000**	3245.5
Long	Arrow	2.149	3.787	**0.0000**	1834.0
Long	Congestion info + arrow + top down view + team spirit	2.016	3.885	**0.0000**	3133.5
Long	Congestion info + arrow + top down view	2.311	4.022	**0.0000**	1720.5
Long	Congestion info + arrow + team spirit	1.940	3.700	**0.0000**	2105.0
Medium	Arrow + top down view	2.708	2.769	0.7852	2168.0
Medium	Arrow + team spirit	2.643	2.946	0.1015	1825.5
Medium	Congestion info + arrow	2.672	3.483	**0.0004**	2298.5
Medium	Arrow + top down view + team spirit	2.677	2.903	0.2906	2122.0
Medium	Arrow	2.723	2.872	0.5057	1185.5
Medium	Congestion info + arrow + top down view + team spirit	2.607	3.246	**0.0045**	2391.0
Medium	Congestion info + arrow + top down view	2.600	3.622	**0.0000**	1503.5
Medium	Congestion info + arrow + team spirit	2.380	3.620	**0.0000**	1924.0
Short	Arrow + top down view	4.754	3.338	**0.0000**	847.0
Short	Arrow + team spirit	4.732	3.321	**0.0000**	465.0
Short	Congestion info + arrow	4.603	2.483	**0.0000**	284.0
Short	Arrow + top down view + team spirit	4.806	3.403	**0.0000**	644.0
Short	Arrow	4.681	3.128	**0.0000**	342.0
Short	Congestion info + arrow + top down view + team spirit	4.787	2.689	**0.0000**	357.5
Short	Congestion info + arrow + top down view	4.667	2.533	**0.0000**	223.0
Short	Congestion info + arrow + team spirit	4.800	2.540	**0.0000**	204.0

Tab. S2D. Effect of information provision on how fans evaluate the route attractiveness (Mann Whitney U test). If information provision makes a significant difference ($p < 0.05$) we marked the corresponding p-Value bold. Through information the long route becomes always more attractive (the means increase), while the short route always becomes less attractive (the means reduce). Also, the medium route becomes more attractive. The mean value always increases, but the difference is not always significant.

Fans

Route	Condition	Route attractiveness (mean value)		p	W
		Prior to info.	*Info. provided*		
Long	Arrow + top down view	2.207	3.550	**0.0000**	9524.0
Long	Arrow + team spirit	2.146	3.553	**0.0000**	8471.5
Long	Congestion info + arrow	1.981	3.505	**0.0000**	8584.5
Long	Arrow + top down view + team spirit	2.125	3.743	**0.0000**	15171.0
Long	Arrow	2.035	3.427	**0.0000**	15846.0
Long	Congestion info + arrow + top down view + team spirit	2.131	3.879	**0.0000**	9755.0
Long	Congestion info + arrow + top down view	2.259	3.897	**0.0000**	11034.0
Long	Congestion info + arrow + team spirit	2.118	3.980	**0.0000**	8914.0
Medium	Arrow + top down view	2.793	3.207	**0.0053**	7424.5
Medium	Arrow + team spirit	2.816	3.078	0.0991	5969.0
Medium	Congestion info + arrow	2.718	3.534	**0.0000**	7383.0
Medium	Arrow + top down view + team spirit	3.029	3.066	0.8674	9352.0
Medium	Arrow	2.909	3.266	**0.0045**	12116.5
Medium	Congestion info + arrow + top down view + team spirit	3.028	3.542	**0.0005**	7214.5
Medium	Congestion info + arrow + top down view	2.974	3.655	**0.0000**	8926.5
Medium	Congestion info + arrow + team spirit	2.922	3.539	**0.0001**	6799.5
Short	Arrow + top down view	4.523	3.414	**0.0000**	2913.5
Short	Arrow + team spirit	4.670	3.786	**0.0000**	3165.0
Short	Congestion info + arrow	4.583	2.806	**0.0000**	1690.5
Short	Arrow + top down view + team spirit	4.669	3.265	**0.0000**	3666.5
Short	Arrow	4.692	3.643	**0.0000**	5235.0
Short	Congestion info + arrow + top down view + team spirit	4.654	2.757	**0.0000**	1507.5
Short	Congestion info + arrow + top down view	4.560	2.784	**0.0000**	2294.0
Short	Congestion info + arrow + team spirit	4.725	2.471	**0.0000**	935.5

Tab. S2E. Route attractiveness prior to information (5-Point Likert scale). We conducted two surveys: one with students & and faculty associates and one with football fans. For each route, we asked them how likely it is that they take the respective route when no route recommendation is provided. Since no information is provided, the message design does not have any effect (see Tab. S2G).

Prior to information

Route	Group	Route attractiveness			
		mean	median	std	n
Long	Fan	2.124	2	0.989	921
Medium	Fan	2.904	3	1.068	921
Short	Fan	4.636	5	0.807	921
Long	Student & faculty associate	2.128	2	1.038	444
Medium	Student & faculty associate	2.631	2	1.042	444
Short	Student & faculty associate	4.732	5	0.700	444

Tab. S2F. Route preference prior to information. For each group, the route attractiveness of the short, medium and long route differ significantly (Dunn's test). In every comparison, the shorter route is preferred ($Z < 0$). We conclude that there is a clear route preference: the short route is favored over the medium route followed by long route (see also the mean values from Tab. S2E).

Comparison of attractiveness	Group	Z	p
Long route and Medium route	Fan	-11.6806	0.0000
Long route and Short route	Fan	-38.0125	0.0000
Medium route and Short route	Fan	-26.3319	0.0000
Long route and Medium route	Student & faculty associate	-5.4089	0.0000
Long route and Short route	Student & faculty associate	-26.3982	0.0000
Medium route and Short route	Student & faculty associate	-20.9893	0.0000

Tab. S2G. Effect of message design on route attractiveness (Kruskal Wallis test). Prior to information (Tab. S2E), the message design has no effect ($p > 0.05$). When information is provided (Tab. S2H), the message design has an effect ($p \leq 0.05$) except for the attractiveness of the long route for the student & faculty associate group.

Information	Route	Group	p	H	df
Prior to information	Fans	Long	4.5498	0.7150	7
Prior to information	Fans	Medium	9.4997	0.2190	7
Prior to information	Fans	Short	6.5020	0.4820	7
Prior to information	Students & faculty associates	Long	0.5950	5.5312	7
Prior to information	Students & faculty associates	Medium	0.8430	3.4222	7
Prior to information	Students & faculty associates	Short	0.3660	7.6299	7
After receiving information	Fans	Long	0.0022	22.42	7
After receiving information	Fans	Medium	0.0000	33.04	7
After receiving information	Fans	Short	0.0000	95.76	7
After receiving information	Students & faculty associates	Long	0.8010	3.811	7
After receiving information	Students & faculty associates	Medium	0.0000	32.38	7
After receiving information	Students & faculty associates	Short	0.0000	45.35	7

Tab. S2H. Route attractiveness when recommending the long route (5-Point Likert scale). We conducted two surveys: one with students & faculty associates (student*) and one with football fans. For each route, we asked them how likely it is that they take the respective route when a route recommendation is provided. The message design always has an effect on the attractiveness of the routes except for the attractiveness of the long route, see Kruskal Wallis tests (see Tab. S2G).

After receiving information

Route	Group	Message design	Route attractiveness			
			mean	median	std	n
Long	Fan	Arrow	3.427	4	1.340	143
Long	Fan	Arrow + team spirit	3.553	4	1.194	103
Long	Fan	Arrow + top down view	3.550	4	1.270	111
Long	Fan	Arrow + top down view + team spirit	3.743	4	1.259	136
Long	Fan	Congestion info + arrow	3.505	4	1.298	103
Long	Fan	Congestion info + arrow + team spirit	3.980	4	1.219	102
Long	Fan	Congestion info + arrow + top down view	3.897	4	1.160	116
Long	Fan	Congestion info + arrow + top down view + team spirit	3.879	4	1.079	107
Medium	Fan	Arrow	3.266	4	1.156	143
Medium	Fan	Arrow + team spirit	3.078	3	1.135	103
Medium	Fan	Arrow + top down view	3.207	4	1.113	111
Medium	Fan	Arrow + top down view + team spirit	3.066	3	1.206	136
Medium	Fan	Congestion info + arrow	3.534	4	1.119	103
Medium	Fan	Congestion info + arrow + team spirit	3.539	4	1.158	102
Medium	Fan	Congestion info + arrow + top down view	3.655	4	1.039	116
Medium	Fan	Congestion info + arrow + top down view + team spirit	3.542	4	1.093	107
Short	Fan	Arrow	3.643	4	1.286	143
Short	Fan	Arrow + team spirit	3.786	4	1.311	103
Short	Fan	Arrow + top down view	3.414	4	1.254	111
Short	Fan	Arrow + top down view + team spirit	3.265	3	1.312	136
Short	Fan	Congestion info + arrow	2.806	2	1.329	103
Short	Fan	Congestion info + arrow + team spirit	2.471	2	1.224	102
Short	Fan	Congestion info + arrow + top down view	2.784	2	1.357	116
Short	Fan	Congestion info + arrow + top down view + team spirit	2.757	2	1.265	107
Long	Student*	Arrow	3.787	4	1.178	47
Long	Student*	Arrow + team spirit	3.696	4	1.190	56
Long	Student*	Arrow + top down view	3.938	4	1.158	65
Long	Student*	Arrow + top down view + team spirit	3.823	4	1.048	62
Long	Student*	Congestion info + arrow	3.862	4	1.146	58
Long	Student*	Congestion info + arrow + team spirit	3.700	4	1.282	50
Long	Student*	Congestion info + arrow + top down view	4.022	4	1.033	45
Long	Student*	Congestion info + arrow + top down view + team spirit	3.885	4	1.305	61
Medium	Student*	Arrow	2.872	2	1.191	47
Medium	Student*	Arrow + team spirit	2.946	3	1.052	56
Medium	Student*	Arrow + top down view	2.769	2	1.129	65
Medium	Student*	Arrow + top down view + team spirit	2.903	3	1.197	62
Medium	Student*	Congestion info + arrow	3.483	4	1.246	58
Medium	Student*	Congestion info + arrow + team spirit	3.620	4	1.276	50
Medium	Student*	Congestion info + arrow + top down view	3.622	4	1.134	45
Medium	Student*	Congestion info + arrow + top down view + team spirit	3.246	4	1.247	61
Short	Student*	Arrow	3.128	3	1.172	47
Short	Student*	Arrow + team spirit	3.321	4	1.162	56
Short	Student*	Arrow + top down view	3.338	3	1.314	65
Short	Student*	Arrow + top down view + team spirit	3.403	3	1.221	62
Short	Student*	Congestion info + arrow	2.483	2	1.047	58
Short	Student*	Congestion info + arrow + team spirit	2.540	2	1.199	50
Short	Student*	Congestion info + arrow + top down view	2.533	2	1.198	45
Short	Student*	Congestion info + arrow + top down view + team spirit	2.689	2	1.218	61
